# Effects of different phosphate lowering strategies in patients with CKD on laboratory outcomes: A systematic review and NMA

**DOI:** 10.1371/journal.pone.0171028

**Published:** 2017-03-01

**Authors:** Nigar Sekercioglu, Argie Angeliki Veroniki, Lehana Thabane, Jason W. Busse, Noori Akhtar-Danesh, Alfonso Iorio, Luciane Cruz Lopes, Gordon H. Guyatt

**Affiliations:** 1 Department of Health Research Methods, Evidence, and Impact, McMaster University, Hamilton, Ontario, Canada; 2 Li Ka Shing Knowledge Institute, St. Michael’s Hospital, Toronto, Ontario, Canada; 3 Department of Pediatrics and Anesthesia, McMaster University, Hamilton, Ontario, Canada; 4 Centre for Evaluation of Medicine, St Joseph's Healthcare—Hamilton, Hamilton, Ontario, Canada; 5 Biostatistics Unit, Father Sean O'Sullivan Research Centre, St Joseph's Healthcare, Hamilton, Ontario, Canada; 6 Population Health Research Institute, Hamilton Health Sciences, Hamilton, Ontario, Canada; 7 The Michael G. DeGroote Institute for Pain Research and Care, McMaster University, Hamilton, Ontario, Canada; 8 Department of Anesthesia, McMaster University, Hamilton, Ontario, Canada; 9 School of Nursing, McMaster University, Hamilton, Ontario, Canada; 10 Department of Medicine, McMaster University, Hamilton, Ontario, Canada; 11 Pharmaceutical Sciences Master Course, University of Sorocaba, UNISO, Sorocaba, Brazil; Universita degli Studi di Perugia, ITALY

## Abstract

**Background:**

Chronic kidney disease-mineral and bone disorder (CKD-MBD), a complication of chronic kidney disease, has been linked to reduced quality and length of life. High serum phosphate levels that result from CKD-MBD require phosphate-lowering agents, also known as phosphate binders. The objective of this systematic review is to compare the effects of available phosphate binders on laboratory outcomes in patients with CKD-MBD.

**Methods:**

Data sources included MEDLINE and EMBASE from January 1996 to April 2016, and the Cochrane Register of Controlled Trials up to April 2016. Teams of two reviewers, independently and in duplicate, screened titles and abstracts and potentially eligible full text reports to determine eligibility, and subsequently abstracted data and assessed risk of bias in eligible randomized controlled trials (RCTs). Eligible trials enrolled patients with CKD-MBD and randomized them to receive calcium-based phosphate binders (delivered as calcium acetate, calcium citrate or calcium carbonate), non-calcium-based phosphate binders (NCBPB) (sevelamer hydrochloride, sevelamer carbonate, lanthanum carbonate, sucroferric oxyhydroxide and ferric citrate), phosphorus restricted diet (diet), placebo or no treatment and reported effects on serum levels of phosphate, calcium and parathyroid hormone.

We performed Bayesian network meta-analyses (NMA) to calculate the effect estimates (mean differences) and 95% credible intervals for serum levels of phosphate, calcium and parathyroid hormone. We calculated direct, indirect and network meta-analysis estimates using random-effects models. We applied the GRADE (Grading of Recommendations, Assessment, Development and Evaluation) approach to rate the quality of evidence for each pairwise comparison.

**Results:**

Our search yielded 1108 citations; 71 RCTs were retrieved for full review and 16 proved eligible. Including an additional 13 studies from a previous review, 29 studies that enrolled 8335 participants proved eligible; 26 trials provided data for quantitative synthesis. Sevelamer, lanthanum, calcium, iron, diet and combinations of active treatments (calcium or sevelamer or lanthanum and combination of calcium and sevelamer) resulted in significantly lower serum phosphate as compared to placebo (moderate to very low quality of evidence). We found no statistically significant differences between active treatment categories in lowering serum phosphate. Sevelamer, lanthanum and diet resulted in lower serum calcium compared to calcium (moderate quality evidence for lanthanum and diet; low quality evidence for Sevelamer). Iron, sevelamer and calcium yielded lower parathyroid hormone levels as compared to lanthanum. Meta-regression analyses did not yield a statistically significant association between treatment effect and trial duration.

**Discussion/Conclusions:**

We found few differences between treatments in impact on phosphate and differences in parathyroid hormone. Relative to calcium, sevelamer, lanthanum and diet showed significant reduction in serum calcium from baseline. Treatment recommendations should be based on impact on patient-important outcomes rather than on surrogate outcomes.

Systematic review registration: PROSPERO CRD-42016032945

## Background

Chronic kidney disease (CKD) has been linked to negative patient outcomes, including mortality, often due to cardiovascular diseases [1–7]. CKD also contributes to comorbid conditions with extra-renal manifestations, such as disturbances of calcium-phosphate homeostasis collectively referred to as CKD mineral and bone disorder (CKD-MBD). CKD-MBD is a systematic disorder that results in adverse bone outcomes (e.g., fractures due to abnormal structure and composition of bones) and cardiovascular outcomes (i.e., cardiovascular calcifications and subsequent cardiovascular events) [2].

In patients suffering from CKD-MBD, clinical practice guidelines suggest maintaining targets for serum phosphate, calcium and parathyroid hormone [8–10]. Dietary restrictions and phosphate binders are commonly used to prevent long-term complications of high serum phosphate (i.e., cardiovascular and soft tissue calcifications) [11–14]. Calcium-based phosphate binders (henceforth referred to as calcium), such as calcium acetate, calcium citrate and calcium carbonate, may lead to positive calcium balance and hypercalcemia [11–14]. Non-calcium-based phosphate binders (NCBPB) include sevelamer, lanthanum and iron (e.g., ferric citrate and sucroferric oxyhydroxide) [11–14]. The combination of calcium carbonate and magnesium carbonate is a new phosphate binding agent [15]. All phosphate binders work in the gastrointestinal system by increasing the excretion of phosphate [16–19].

Previous systematic reviews have addressed the impact of alternative interventions for CKD-MBD on outcomes of important to patients, including all-cause mortality [20, 21]. Jamal et al. conducted a systematic review and explored the effectiveness of calcium versus NCBPBs in patients with CKD-MBD. The results suggest higher mortality with calcium binders than with NCBPBs [21].

Consistent with Jamal’s review, our systematic review and network meta-analysis (NMA) found that calcium versus sevelamer resulted in higher mortality among CKD-MBD patients [22]. Our NMA results were congruent with our conventional meta-analysis in terms of the direction, magnitude and statistical significance of the effects of phosphate binders on mortality [22]. Although not statistically significant, conventional meta-analysis results also showed higher cardiovascular mortality and hospitalization with calcium binders relative to NCBPBs [22].

The association between drug effects on laboratory outcomes and patient survival has been explored with mixed results [23, 24]. Nevertheless, the impact of the interventions on patient-important outcomes is likely to be mediated through effects on target physiological variables: phosphate, calcium, and parathyroid hormone. This line of thinking is consistent with the majority of clinical practice guidelines, which base their recommendations regarding the management of CKD-MBD on laboratory outcomes [8, 25–28].

Knowledge of the impact of interventions on these surrogate outcomes may provide insight into understanding of the results of randomized controlled trials (RCTs) that address patient-important outcomes, and might provide clues regarding the comparative effectiveness of NCBPB agents on patient-important outcomes, currently unestablished. The objective of this study was therefore to systematically review and synthesize evidence from RCTs addressing the effectiveness of phosphorus restricted diet (diet) and different phosphate binders on serum levels of phosphate, calcium and parathyroid hormone by combining direct and indirect estimates in a NMA. We updated the Jamal systematic review [21] and applied the Grading of Recommendations, Assessment, Development and Evaluation (GRADE) approach to assess the quality of evidence on an outcome-by-outcome basis.

In our previous review, we included patient-important outcomes using the frequentist framework. In this study, we only assessed surrogate outcomes (calcium, phosphate and parathyroid hormone). When conducting meta-regression, the Bayesian framework provides a common coefficient for all treatment comparisons. It is not possible to produce a common coefficient in the frequentist framework. In this study, we were able to perform meta-regression, since we used the Bayesian framework.

## Methods

We registered our protocol on PROSPERO (CRD42016032945) and adhered to the Preferred Reporting Items for Systematic Review and Meta-analysis for Network Meta-analysis (PRISMA NMA) guidelines in drafting our manuscript (File A in the S1 Supporting Information File) [29].

### Eligibility criteria

We included studies that: (1) enrolled patients with CKD, defined as an estimated glomerular filtration rate <60 ml/min/1.73 m^2^, including dialysis and non-dialysis CKD patients; (2) randomized patients to a diet or phosphate binder versus a control; (3) reported at least one of the following outcomes: serum phosphate, calcium or parathyroid hormone; and (4) had a minimum follow-up of 4 weeks. Phosphate binders included calcium (calcium acetate, calcium citrate or calcium carbonate) or NCBPBs (sevelamer hydrochloride, sevelamer carbonate, lanthanum carbonate, sucroferric oxyhydroxide or ferric citrate). A control included placebo or no intervention. We excluded non-randomized controlled trials, observational studies and conference abstracts.

### Data sources and search strategy

We used the search of MEDLINE and EMBASE that we performed in our recently published review [22] which was based on a prior review [21] and updated search for the subsequent period. We scanned references of all prior systematic reviews and meta-analyses as well as all eligible primary studies for additional relevant articles [21]. We established search alerts for monthly notifications and repeated the search before submission to identify any new relevant trials from the MEDLINE and EMBASE databases. File B in the S1 Supporting Information File presents the full search strategy.

### Study selection

Teams of two reviewers independently screened each title and abstract. If either reviewer identified a citation as potentially relevant, we obtained the full text of the article. Two reviewers independently determined the eligibility of all studies that underwent full text evaluation and resolved discrepancies by discussion.

### Data abstraction

We extracted study data using a customized data collection form accompanied by a detailed instruction manual. Two independent reviewers abstracted the following information from each study: (1) author, (2) year of publication, (3) summary of baseline characteristics of the participants, (4) trial duration, and (5) serum levels of phosphate, parathyroid hormone or calcium. We recorded the last measurement if multiple measurements were provided during the follow-up period.

### Risk of bias of included studies

Two reviewers used a modified version of the Cochrane risk for bias tool in order to assess the risk of bias on the basis of randomization, allocation concealment, blinding, incomplete outcome data, selective reporting (by comparing the methods and results sections of the manuscript) as well as stopping early for benefit [30]. Reviewers chose among response options of “definitely yes”, “probably yes”, “probably no”, and “definitely no” for each of the domains, with “definitely yes” and “probably yes” ultimately assigned low risk of bias and “definitely no” and “probably no” assigned high risk of bias [31].

### Quality assessment of evidence

We assessed the quality of evidence in effect estimates for each outcome as high, moderate, low or very low using the GRADE rating system [32] in which RCTs begin as high quality evidence, but may be rated down by one or more of five categories of limitations [31]: risk of bias, precision, consistency, directness or publication bias [33].

After considering these reasons for rating down, we judged the overall confidence in estimates of effect for change in serum phosphate, calcium and parathyroid hormone from baseline for each pairwise comparison as follows: ‘high’ quality of evidence (we are very confident that the true effect lies close to that of the estimate of the effect); ‘moderate’ quality of evidence (we are moderately confident in the effect estimate and the true effect is likely to be close to the estimate of the effect, but there is a possibility that it is substantially different); ‘low’ quality of evidence (our confidence in the effect estimate is limited and the true effect may be substantially different from the estimate of the effect); and ‘very low’ quality of evidence (we have very little confidence in the effect estimate and the true effect is likely to be substantially different from the estimate of effect) [31].

We also applied the GRADE methodology to rate the confidence of indirect effect estimates. In relation to the treatment comparisons, we visually examined the network graphs and identified first order (one intervention connecting to two interventions, also called a single common comparator) and higher order loops (more than one interventions connecting to the two interventions). The quality of evidence rating for the indirect comparisons informing each pairwise comparison was the lower of the ratings of quality for the two direct estimates contributing to the first order loop. For instance, if one contributing direct comparison was rated as low and other rated as moderate evidence, we rated the quality of indirect evidence as low [34]. In the absence of a first order loop, a higher order loop was used to rate quality of evidence. In a higher order loop, we identified all contributing comparisons and quality of evidence in each comparison. The quality of evidence rating for the indirect comparisons in each higher order loop was the lower of the ratings of quality for the direct estimates contributing to the higher order loop.

In the GRADE system for NMA, indirect effect estimates may be further rated down for intransitivity. The transitivity assumption implies similarity of trials in terms of population, intervention (type and dosing frequency), settings and trial methodology across the treatment comparisons included in the network. If the transitivity assumption was deemed to be violated, we planned to rate down the indirect comparison by one further level (if possible), as well as to explore this meta-regression analysis. Trial duration was the only effect modifier that we assessed for this assumption. All other potential effect modifiers, indeed, were not assessed using meta-regression due to unavailability of the data, such as mean age.

If both direct and indirect evidence were available, the NMA quality rating came from the higher of the two. If there was direct evidence, but no indirect evidence because of no closed loop or if there was a closed loop formed by a multi-arm trial, the NMA was graded according to the direct evidence. If there was no direct evidence, the NMA received the GRADE assessment of the indirect estimate.

We also considered coherence (degree of consistency between direct and indirect effect estimates) in our final quality rating of network estimates. We visually examined the magnitude of the difference between direct and indirect effect estimates and the extent to which their confidence intervals overlapped. We planned to rate down the quality of the NMA effect if we found meaningfully large incoherence. We calculated indirect effect estimates using the node-splitting approach [35]. We have calculated the credible intervals for the between-study variance in every analysis. We used the design-by-treatment interaction model that provides an omnibus test for loop and design inconsistency in the entire network [36, 37]. Hence, we provide the chi-square test and the p-value along with it.

If the NMA evidence was substantially more precise than the higher quality of the direct or indirect estimates, we rated that estimate up due to improved precision.

We used a funnel plot to explore publication bias for comparisons with more than 10 studies in the direct comparisons. Asymmetrical funnel plots indicate reporting biases due to publication bias or small-study effect [38, 39]. However, the visual inspection for the assessment of publication bias is subjective.

In summary, the quality of evidence for each pairwise network comparison included assessment of transitivity (similarity between populations, interventions, comparators and outcomes of trials in the direct comparisons that contribute to the indirect comparison estimate); coherence (similarity between direct and indirect effect estimates); and homogeneity (similarity of effect estimates between trials in direct comparisons).

### Data synthesis and statistical analysis

We used aggregate data (i.e., summary point estimates for all patients included in each study) to perform pairwise and network meta-analyses. In our conventional meta-analysis, we calculated pooled mean differences (MD) and the associated 95% credible intervals (CrIs) for each outcome using random-effects models in a Bayesian framework.

For Bayesian analyses, we used non-informative normal priors for means and a half-normal prior distribution for the between-study standard deviation (τ~N(0,1), τ>0). Posterior distributions were produced using Markov chain Monte Carlo methods. Two sets of initial values were produced for each chain with 100,000 iterations. We planned to increase the number of iterations if the data did not converge. The first 10,000 iterations were discarded as burn-in and a thinning of 10 was also applied [40].

We employed fixed-effect and random-effects models and compared model fit and parsimony using the deviance information criterion (DIC). We reported our final results based on random-effect models because they indicated lower DIC values.

For each outcome, we reported the pooled MD and associated 95% CrIs based on the posterior distributions of Bayesian NMAs. We also calculated predictive intervals (PrIs) to capture the magnitude of the between-study variance for each outcome per phosphate binder. PrIs present the intervals within which we would expect the treatment effect of a future study to lie [30]. For each outcome, we present network graphs, the NMA effect estimates and ranking of treatments according to their effectiveness using the surface under the cumulative ranking probabilities (SUCRA) curve [41]. We present SUCRA values from all outcomes in a single diagram using a rank-heat plot [42, 43].

Modified Gelman-Rubin statistics and graphical assessment of trace plots were used to examine model convergence. The analysis was performed using OpenBUGS 3.2.3 (*MRC Biostatistics unit*, *Cambridge*, *UK*), which generated inferences using the Gibbs sampler. We performed analysis for indirect estimates in R studio using the *gemtc* package [44]. We assessed consistency using the *network* command in Stata (StataCorp. 2013. Stata Statistical Software: Release 13. College Station, TX: StataCorp LP) [37].

We employed a network meta-regression in order to examine an association between treatment effect using trial duration as a continuous variable measured in months. We also conducted a subgroup analysis for dialysis versus non-dialysis patients.

## Results

### Trial identification

**Fig 1:**
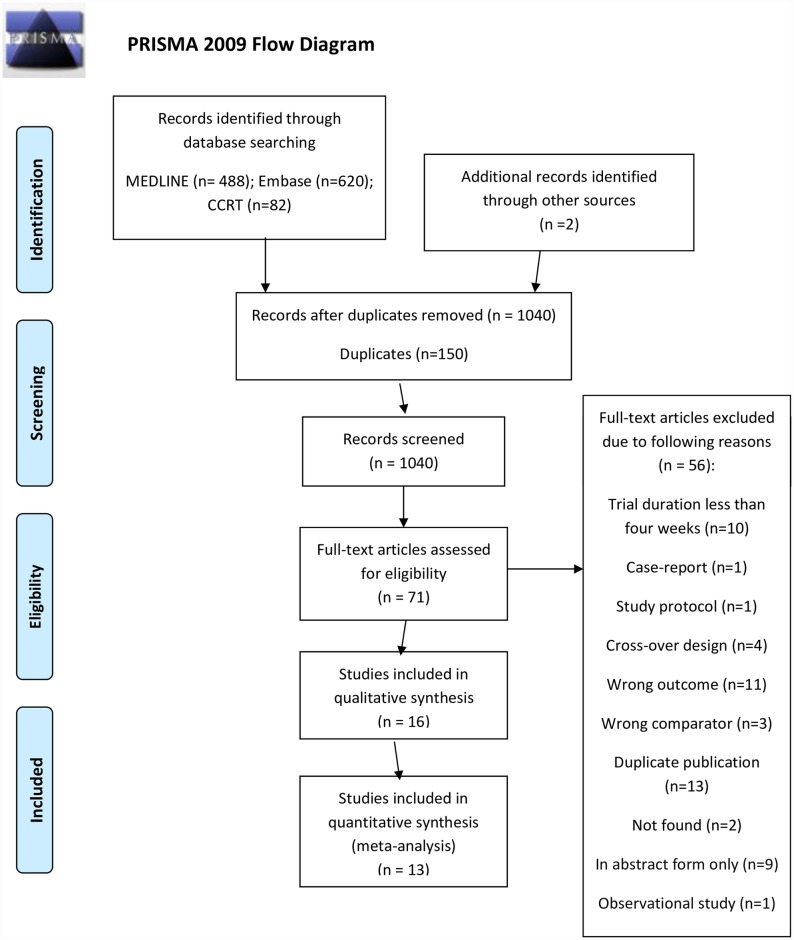
PRISMA flow diagram.

Our updated search yielded 1108 citations, of which 71 were retrieved for full review; 16 RCTs including 3576 patients proved eligible (Fig 1). We included 13 RCTs from the previous systematic review [21] for a total of 29 eligible studies with 8397 participants; 26 provided data (n = 6760) that allowed inclusion in our quantitative synthesis (Fig 1).

### Trial and population characteristics

Table A in the S1 Supporting Information File presents the characteristics of all eligible studies [45–73]. Eight of the twenty-nine studies (28%) included non-dialysis patients. Year of publication ranged from 2002 to 2015. A total of 11 trials were multinational and all were multi-centre. The mean age of participants ranged from 47 to 69. Table B in the S1 Supporting Information File represents treatment codes, treatment categories and abbreviations used in the analysis while Table C shows treatment comparisons, number of studies and number of patients for phosphate outcome.

### Assessment of consistency between direct and indirect estimates

The omnibus test of consistency between direct and indirect estimates did not approach significance for any of the three outcomes (degrees of freedom [d.f.] = 3, chi-square test = 1.76, p = 0.62 for phosphate, d.f. = 6, chi-square test = 3.77, p = 0.70 for calcium and d.f. = 6, chi-square test = 6.35, p = 0.38 for parathyroid hormone).

### Assessment of risk of bias in individual studies and quality of evidence in conventional pair-wise meta-analyses

**Fig 2:**
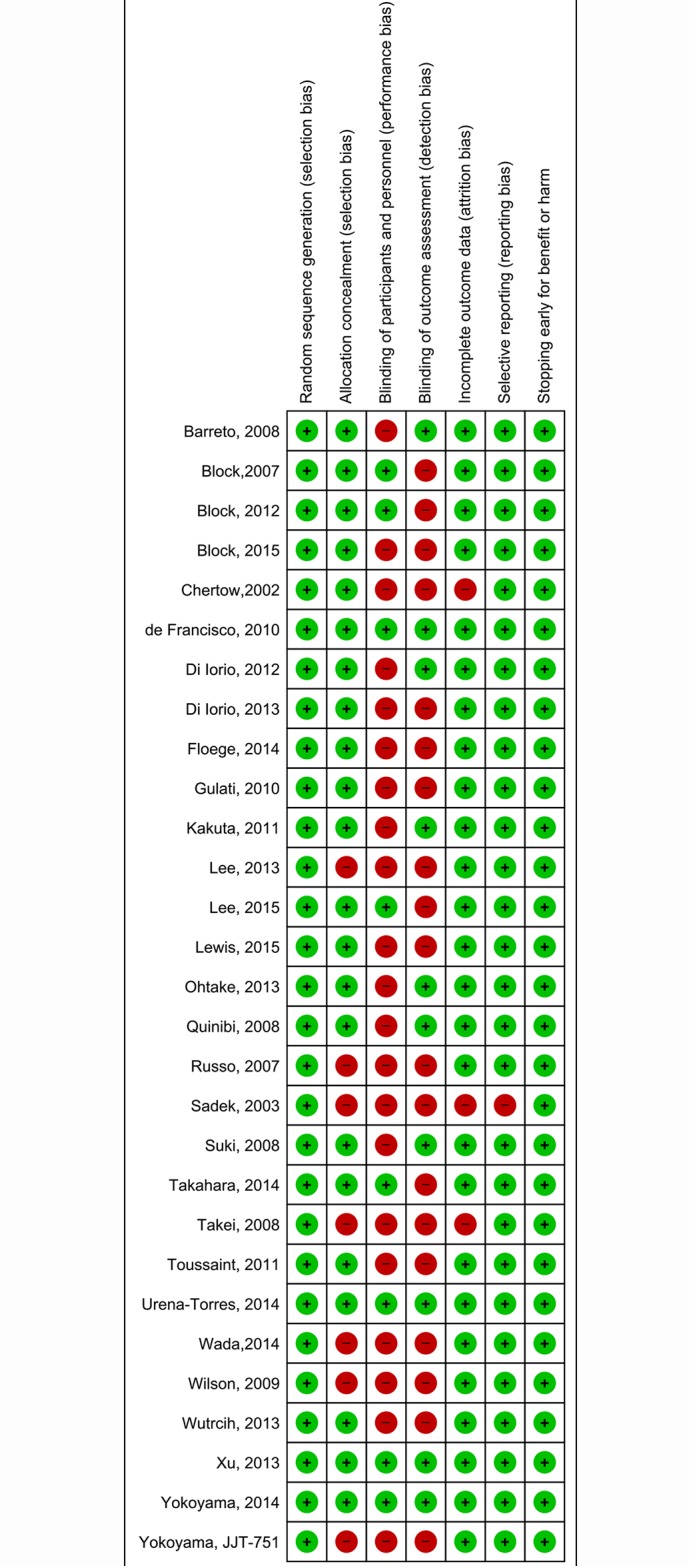
Risk of bias assessment for surrogate outcomes.

Our assessment indicated low risk of bias for missing data and selective reporting in about 95% of the trials; blinding was adequate in only about 25% of the trials (Fig 2). Of the ten pairwise comparisons for phosphate, we classified one as high quality, four as moderate quality, three as low quality and two very low quality (Tables D, E and F in the S1 Supporting Information File). Of the eleven pairwise comparisons for calcium, we classified five as high quality, four as moderate quality, one as low quality and one as very low quality (Tables G, H and I in the S1 Supporting Information File). Of the twelve pairwise comparisons for parathyroid hormone, we classified seven as high quality, two as moderate quality, two as low quality and one as very low quality (Tables K, l and M in the S1 Supporting Information File).

### Direct treatment comparisons from conventional pair-wise meta-analysis

Lanthanum was associated with significant reductions in serum phosphate level as compared to placebo (-0.88 mg/dl [95% CrI, -1.63 to -0.84]) as was iron (-1.43 mg/dl [95% CrI, -2.20 to -0.70]) (Table D in the S1 Supporting Information File). In the comparison of diet and calcium, the results indicated significant lower phosphate levels with diet (-0.80 mg/dl [95% CrI, -1.43 to -0.18]). No other differences reached statistical significance (Table F in the S1 Supporting Information File).

Reductions in serum calcium were observed with sevelamer vs. diet (-0.60 mg/dl [95% CrI, -0.74 to -0.46] and sevelamer vs calcium (-0.30 mg/dl [95% CrI, -0.08 to -0.52] (Table G in the S1 Supporting Information File). No other MDs achieved statistical significance.

All phosphate binder comparisons except calcium vs. sevelamer, lanthanum vs. sevelamer, diet vs. sevelamer achieved significantly different mean reduction in serum parathyroid hormone levels (Table K in the S1 Supporting Information File). Iron, as compared to sevelamer, led to greater parathyroid hormone reduction (-8 pg/ml [95% CrI, -17 to -0.52]). Calcium was associated with significant reductions in serum parathyroid hormone as compared to placebo (-67 pg/ml [95% CrI, -131 to—4]) as was lanthanum (-45 mg/dl [95% CrI, -83 to -11]).

### Network meta-analysis: Phosphate

**Fig 3:**
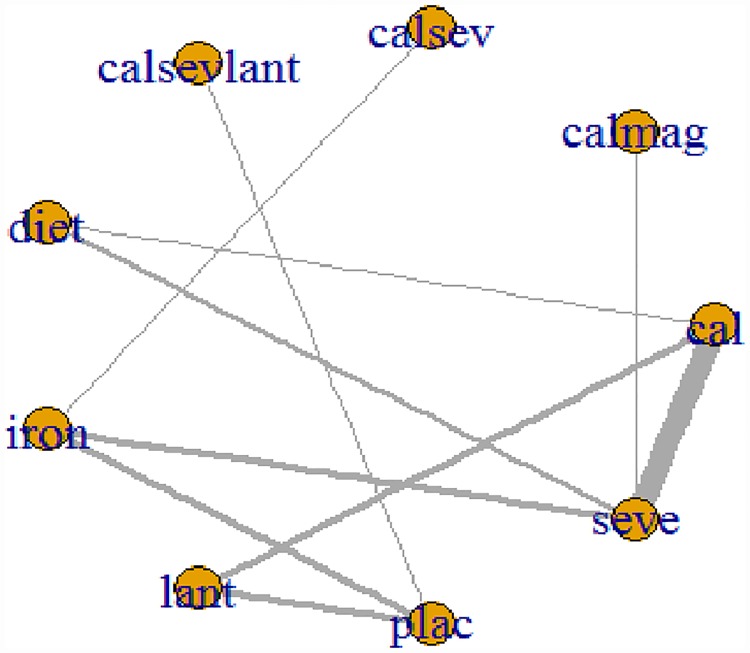
Network of clinical trials of phosphate binders in patients with chronic kidney disease: outcome mean change from baseline in serum phosphate concentration. Netplot of effectiveness outcome for mean phosphate reduction at the end of the study period. Network of randomized controlled trials comparing different phosphate binders for mean change in serum phosphate. Lines connect different phosphate binder categories with direct evidence. The width of lines correlates the number of RCTs for each direct comparison while the size of the nodes correlates with the total sample size. Abbreviations: cal: calcium; calmag: calcium and magnesium; calsev: calcium and Sevelamer; calsevlant: calcium or sevelamer or lanthanum;lant: lanthanum; seve: Sevelamer.

**Fig 4:**
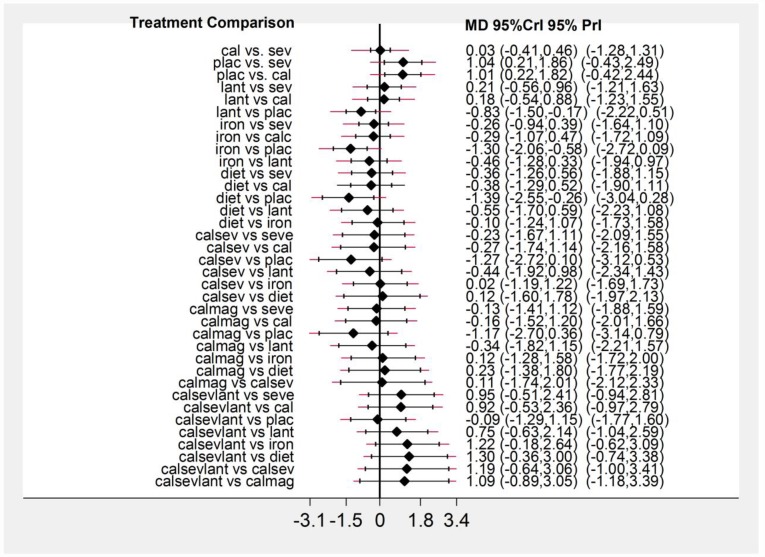
Network meta-analysis results for serum phosphate. Forest plot of effectiveness outcome for mean phosphate reduction at the end of the study period. MD: Mean difference; Crl: Credible interval; PrI: predictive intervals.

Fig 3 presents the network plot for phosphate. Of the twenty-six RCTs evaluating nine treatments or treatment combinations of phosphate binders from seven pharmacological and non-pharmacological interventions reported data on the change in serum phosphate levels. Fig 4 displays a forest plot of the mean changes and 95% CrIs and 95% and PrIs of reduction in serum phosphate levels for all pairwise comparisons from the network.

Relative to placebo, sevelamer, lanthanum, calcium, iron, diet and combination of active treatments (calcium or sevelamer or lanthanum and combination of calcium and sevelamer) showed significant reduction in serum phosphate level (Table 1). No other pairwise comparisons showed statistically significant differences, except iron versus combination of sevelamer or calcium or lanthanum category (1.31 mg/dl [95% CrI, 0.01 to 2.67], [95% PrI, -0.43 to 3.14]) (moderate quality evidence). Of the 29 comparisons that failed to reach statistical significance in the network estimate, we classified six as moderate quality, ten as low quality, and 13 as very low quality evidence. We assessed publication bias by a funnel plot which did not indicate asymmetry (Fig A in the S1 Supporting Information File).

**Table 1:** Direct, indirect, and NMA estimates of phosphate with 95% credible intervals and GRADE assessments from each pairwise comparison within the phosphate-binder network.

Treatment Comparison		Direct estimate; MD (95% CrI)	Quality of evidence	Indirect estimate; MD (95% CrI)	Quality of evidence	NMA estimate; MD (95% CrI)	Quality of evidence
Sevelamer	Calcium	0.05 (-0.36, 0.46)	Low	0.11 (-1.40, 1.61)	Low	0.09 (-0.29, 0.47)	Low
Sevelamer	Placebo	NA	NA	1.13 (0.35, 1.90)	Very Low	1.13 (0.35, 1.90)	Very Low
Calcium	Placebo	NA	NA	1.03 (0.26, 1.81)	Low	1.03 (0.26, 1.81)	Low
Lanthanum	Sevelamer	NA	NA	0.24 (-0.49, 0.98)	Low	0.24 (-0.49, 0.98)	Low
Lanthanum	Calcium	0.15 (-0.69, 0.98)	Low	0.17 (-1.20, 1.56)	Very Low	0.15 (-0.54, 0.85)	Low
Lanthanum	Placebo	-0.87 (-1.6, -0.14)	Moderate	-0.90 (-2.34, 0.52)	Very Low	-088 (-1.52, -0.25)	Moderate
Sevelamer	Iron	-0.28 (-1.06, 0.45)	Very Low	-0.31 (-1.77,1.10)	Low	-0.28 (-0.95, 0.34)	Low
Iron	Calcium	NA	NA	-0.38 (-1.09, 0.31)	Very Low	-0.38 (-1.09, 0.31)	Very Low
Iron	Placebo	-1.49 (-2.2, -0.69)	Moderate	-1.42 (-2.85, 0)	Very Low	-1.41 (-2.07, -0.79)	Moderate
Iron	Lanthanum	NA	NA	-0.53 (-1.30, 0.21)	Very Low	-0.53 (-1.30, 0.21)	Very Low
Sevelamer	Diet	-0.20 (-1.12, 0.71)	Very Low	Closed loop formed by a multi-arm trial; not estimated	Not available	-0.24 (-1.08, 0.58)	Very Low
Calcium	Diet	-0.79 (-1.42, -0.17)	High	0.42 (-0.89,1.75)	Very Low	-0.33 (-1.22, 0.54)	Moderate^1^
Diet	Placebo	NA	NA	-1.37 (-2.5, -0.26)	Very Low	-1.37 (-2.5, -0.26)	Very Low
Lanthanum	Diet	NA	NA	-0.49 (-1.58, -0.59)	Low	-0.49 (-1.58, -0.59)	Low
Iron	Diet	NA	NA	0.04 (-0.9, 1.1)	Very Low	0.04 (-0.9, 1.1)	Very Low
Sevelamer	Calsev	NA	NA	-0.26 (-1.62, 1.05)	Very Low	-0.26 (-1.62, 1.05)	Very Low
Calcium	Calsev	NA	NA	-0.36 (-1.75, 0.98)	Very Low	-0.36 (-1.75, 0.98)	Very Low
Placebo	Calsev	NA	NA	-1.39 (-2.76, -0.08)	Moderate	-1.39 (-2.76, -0.08)	Moderate
Lanthanum	Calsev	NA	NA	-0.51 (-1.93, 0.87)	Moderate	-0.51 (-1.93, 0.87)	Moderate
Iron	Calsev	0.01 (-0.003 to 0.04)	Moderate	No closed loop; not estimated	Not available	0.02 (-1.15, 1.19)	Moderate
Calsev	Diet	NA	NA	-0.02 (-1.61, 1.53)	Very Low	-0.02 (-1.61, 1.53)	Very Low
Sevelamer	Calmag	-0.17 (-0.59 to 0.23)	Low	No closed loop; not estimated	Not available	-0.18 (-1.42, 1.05)	Low
Calmag	Calcium	NA	NA	-0.27 (-1.57, 1.03)	Low	-0.27 (-1.57, 1.03)	Low
Calmag	Placebo	NA	NA	-1.31 (-2.77, 0.14)	Low	-1.31 (-2.77, 0.14)	Low
Calmag	Lanthanum	NA	NA	-0.43 (-1.87, 1.02)	Low	-0.43 (-1.87, 1.02)	Low
Calmag	Iron	NA	NA	0.10 (-1.27, 1.52)	Very Low	0.10 (-1.27, 1.52)	Very Low
Calmag	Diet	NA	NA	0.06 (-1.43, 1.56)	Very Low	0.06 (-1.43, 1.56)	Very Low
Calmag	Calsev	NA	NA	0.08 (-1.72, 1.93)	Very Low	0.08 (-1.72, 1.93)	Very Low
Calsevlant	Sevelamer	NA	NA	1.03 (-0.37, 2.44)	Very Low	1.03 (-0.37, 2.44)	Very Low
Calsevlant	Calcium	NA	NA	0.93 (-0.46, 2.35)	Low	0.93 (-0.46, 2.35)	Low
Calsevlant	Placebo	-0.09 (-0.23, 0.03)	Moderate	No closed loop; not estimated	Not available	-0.09 (-1.28, 1.07)	Moderate
Calsevlant	Lanthanum	NA	NA	0.78 (-0.55, 2.13)	Moderate	0.78 (-0.55, 2.13)	Moderate
Calsevlant	Iron	NA	NA	1.31 (0.01, 2.67)	Moderate	1.31 (0.01, 2.67)	Moderate
Calsevlant	Diet	NA	NA	1.27 (-0.34, 2.91)	Very Low	1.27 (-0.34, 2.91)	Very Low
Calsevlant	Calsev	NA	NA	1.29 (-0.45, 3.1)	Moderate	1.29 (-0.45, 3.1)	Moderate
Calsevlant	Calmag	NA	NA	1.21 (-0.65, 3.09)	Very Low	1.21 (-0.65, 3.09)	Very Low

^1^Rated down for incoherence.

Abbreviations: CrI: Credible interval; MD: Mean difference; calmag: calcium and magnesium; calsev: calcium and sevelamer; calsevlant: calcium or sevelamer or lanthanum; NA: not available.

**Fig 5:**
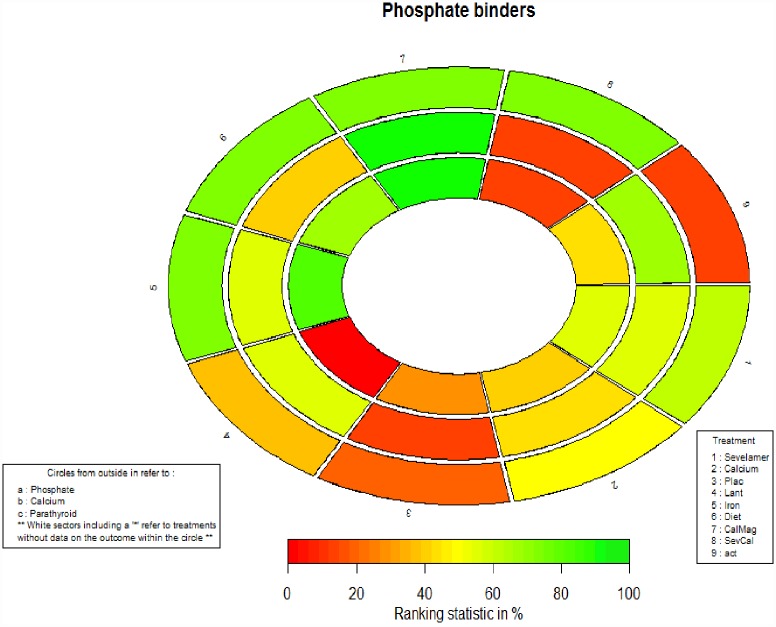
Rank-heat plot of the phosphate binder network for laboratory outcomes.

SUCRA ranking suggested diet as the optimal treatment for reducing serum phosphate (SUCRA, 0.75; 95% CrI, 0.25 to 1.00) (Fig 5). However, credible intervals of the SUCRA value were large. The between-study variance was 0.33 (95% CrI, 0.15 to 0.76) (Table N in the S1 Supporting Information File).

### Network meta-analysis: Calcium

**Fig 6:**
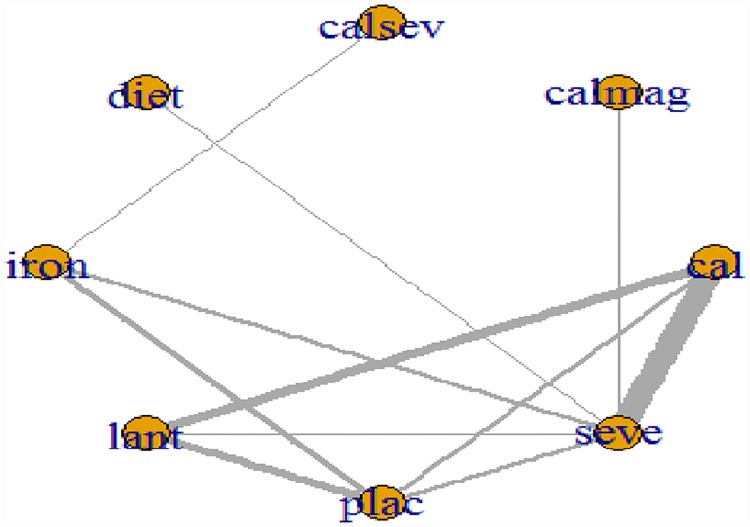
Network of clinical trials of phosphate binders in patients with chronic kidney disease: outcome mean change from baseline in serum calcium concentration. Netplot of effectiveness outcome for mean calcium reduction at the end of the study period. Network of randomized controlled trials comparing different phosphate binders for mean change in serum calcium. Lines connect different phosphate binder categories with direct evidence. The width of lines correlates the number of RCTs for each direct comparison while the size of the nodes correlates with the total sample size. Abbreviations: cal: calcium; calmag: calcium and magnesium; calsev: calcium and Sevelamer; Lant: lanthanum; seve: Sevelamer.

Fig 6 presents the network plot for calcium is depicted in Fig 6. Twenty-six RCTs evaluating eight treatments or treatment combinations of phosphate binders from seven pharmacological and non-pharmacological categories reported data on the change in calcium level from baseline.

**Fig 7:**
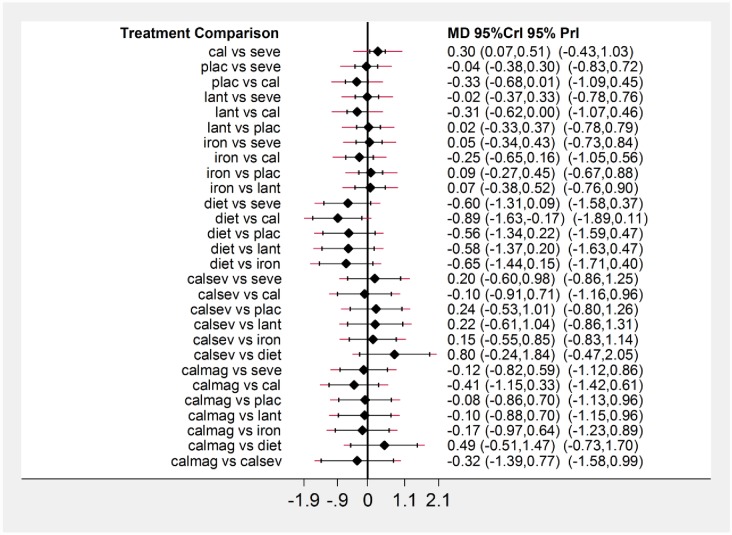
Network meta-analysis results for serum calcium. Forest plot of effectiveness outcome for mean calcium reduction at the end of the study period; MD: Mean difference; Crl: Credible interval; PrI: predictive intervals.

Fig 7 displays a forest plot of the mean changes and 95% CrIs and 95% PrIs of reduction in serum calcium levels for all pairwise comparisons from the network. Relative to calcium, sevelamer, lanthanum and diet showed significant reduction in serum calcium from baseline (-0.30 mg/dl [95% CrI, -0.51 to -0.07] for sevelamer vs. calcium; -0.31 mg/dl [95% CrI, -0.62 to 0] for lanthanum vs. calcium; -0.89 mg/dl [95% CrI, -1.63 to -0.17] for diet vs. calcium) (in comparisons with calcium moderate quality evidence for lanthanum and diet; low quality evidence for sevelamer). There was no statistically significant difference between other drug categories (Table 2).

**Table 2:** Direct, indirect, and NMA estimates of calcium with 95% credible intervals and GRADE assessments from each pairwise comparison within the phosphate-binder network.

Treatment Comparison		Direct estimate; MD (95% CrI)	Quality of evidence	Indirect estimate; MD (95% CrI)	Quality of evidence	NMA estimate; MD (95% CrI)	Quality of evidence
Calcium	Sevelamer	0.30 (0.08 to 0.51)	Moderate	0.08 (-0.82 to 0.97)	Moderate	0.29 (0.07 to 0.51)	Low^1^
Sevelamer	Placebo	0.10 (-0.39 to 0.59)	High	-0.24 (-0.73 to 0.24)	Low	-0.03 (-0.37 to 0.29)	High
Placebo	Calcium	-0.01 (-0.5 to 0.5)	Low	-0.60 (-1.08 to -0.11)	Moderate	-0.33 (-0.67 to 0.01)	Moderate
Sevelamer	Lanthanum	-0.09 (-0.33 to 0.13)	Moderate	0 (-0.42 to 0.41)	Moderate	-0.01 (-0.36 to 0.32)	Moderate
Lanthanum	Calcium	-0.33 (-0.67 to 0.01)	Moderate	-0.20 (-0.90 to 0.53)	Low	-0.31 (-0.62 to 0)	Moderate
Lanthanum	Placebo	0.07 (-0.33 to 0.48)	High	-0.10 (-0.78 to 0.57)	Low	0.02 (-0.33 to 0.36)	High
Sevelamer	Iron	-0.15 (-0.64 to 0.34)	Very Low	0.30 (-0.28 to 0.87)	High	0.04 (-0.33 to 0.43)	High
Iron	Calcium	NA	NA	-0.24 (-0.65 to 0.16)	Very Low	-0.24 (-0.65 to 0.16)	Very Low
Iron	Placebo	0.22 (-0.18 to 0.62)	High	-0.24 (-0.88 to 0.41)	Very Low	0.09 (-0.27 to 0.45)	High
Iron	Lanthanum	NA	NA	0.06 (-0.37 to 0.51)	Very Low	0.06 (-0.37 to 0.51)	Very Low
Sevelamer	Diet	-0.60 (-0.74 to -0.45)	Moderate	No closed loop	Not available	-0.6 (-1.3 to 0.10)	Moderate
Diet	Calcium	NA	NA	-0.89 (-1.62 to -0.15)	Moderate	-0.89 (-1.62 to -0.15)	Moderate
Placebo	Diet	NA	NA	-0.56 (-1.33 to 0.22)	Moderate	-0.56 (-1.33 to 0.22)	Moderate
Lanthanum	Diet	NA	NA	-0.58 (-1.36 to 0.20)	Moderate	-0.58 (-1.36 to 0.20)	Moderate
Iron	Diet	NA	NA	-0.64 (-1.44 to 0.15)	Very Low	-0.64 (-1.44 to 0.15)	Very Low
Sevelamer	Calsev	NA	NA	0.20 (-0.59 to 0.98)	Very Low	0.20 (-0.59 to 0.98)	Very Low
Calcium	Calsev	NA	NA	-0.09 (-0.89 to 0.70)	Very Low	-0.09 (-0.89 to 0.70)	Very Low
Placebo	Calsev	NA	NA	0.23 (-0.54 to 1.01)	High	0.23 (-0.54 to 1.01)	High
Lanthanum	Calsev	NA	NA	0.21 (-0.60 to 1.04)	Very Low	0.21 (-0.60 to 1.04)	Very Low
Iron	Calsev	0.15 (0.13 to 0.16)	High	No closed loop	Not available	0.15 (-0.54 to 0.84)	High
Diet	Calsev	NA	NA	0.80 (-0.26 to 1.86)	Very Low	0.80 (-0.26 to 1.86)	Very Low
Sevelamer	Calmag	-012 (-0.26 to 0.02)	High	No closed loop	Not available	-0.12 (-0.82 to 0.58)	High
Calcium	Calmag	NA	NA	-0.41 (-1.14 to 0.32)	Moderate	-0.41 (-1.14 to 0.32)	Moderate
Placebo	Calmag	NA	NA	-0.08 (-0.86 to 0.70)	High	-0.08 (-0.86 to 0.70)	High
Lanthanum	Calmag	NA	NA	-0.10 (-0.87 to 0.68)	Moderate	-0.10 (-0.87 to 0.68)	Moderate
Iron	Calmag	NA	NA	-0.17 (-0.96 to 0.63)	Very Low	-0.17 (-0.96 to 0.63)	Very Low
Diet	Calmag	NA	NA	0.47 (-0.50 to 1.47)	Moderate	0.47 (-0.50 to 1.47)	Moderate
Calmag	Calsev	NA	NA	-0.32 (-1.37 to 0.74)	Very Low	-0.32 (-1.37 to 0.74)	Very Low

^1^Rated down for incoherence.

Abbreviations: CrI: Credible interval; MD: Mean difference; calmag: calcium and magnesium; calsev: calcium and sevelamer; calsevlant: calcium or sevelamer or lanthanum.

Of the 25 comparisons that failed to reach statistical significance in the network estimate, we classified eight as high quality, eight as moderate quality and nine as very low quality evidence (Table 2).

Patients treated with diet had a higher likelihood of reduction in serum calcium as compared to those treated with other treatment categories (SUCRA, 1; 95% CrI, 0.29 to 1.00) (Fig 5). However, credible intervals of the SUCRA value were large. The between-study variance was 0.11 (95% CrI, 0.06 to 0.24) (Table N in the S1 Supporting Information File).

### Network meta-analysis: Parathyroid hormone

**Fig 8:**
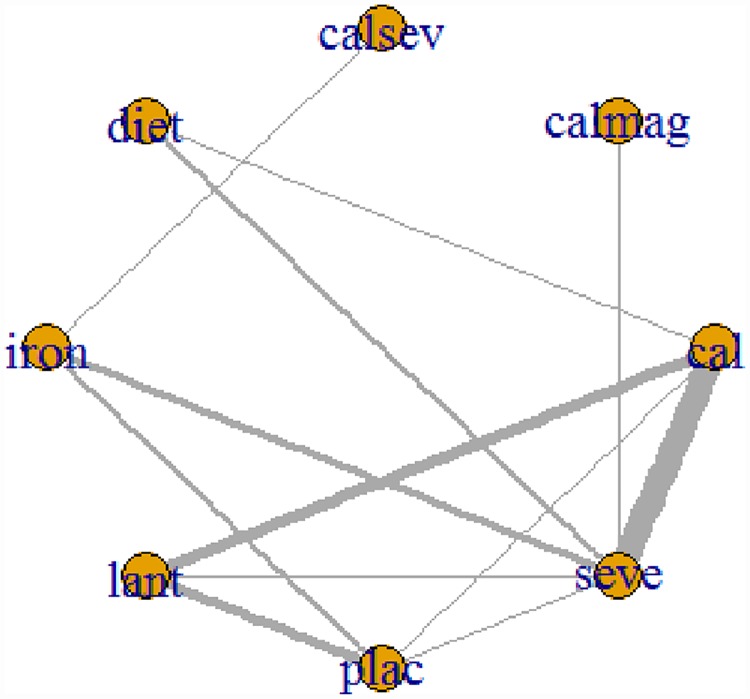
Network of clinical trials of phosphate binders in patients with chronic kidney disease: outcome mean change from baseline in serum parathyroid hormone concentration. Netplot of effectiveness outcome for mean parathyroid hormone reduction at the end of the study period. Network of randomized controlled trials comparing different phosphate binders for mean change in serum parathyroid hormone Lines connect different phosphate binder categories with direct evidence. The width of lines correlates the number of RCTs for each direct comparison while the size of the nodes correlates with the total sample size. Abbreviations: cal: calcium; calmag: calcium and magnesium; calsev: calcium and Sevelamer; Lant: lanthanum; seve: Sevelamer.

Fig 8 presents the network plot for parathyroid hormone. Twenty-six RCTs evaluating eight treatments or treatment combinations of phosphate binders from seven pharmacological and non-pharmacological categories reported data on the change in serum parathyroid hormone level from baseline. Fig 8 displays a forest plot of the mean changes and 95% CrIs and 95% PrIs of reduction in serum parathyroid hormone levels for all pairwise comparisons from the phosphate-binder network.

In individual interventions tested, iron, diet, sevelamer and calcium yielded lower parathyroid hormone levels as compared to lanthanum. Iron was more effective in reducing parathyroid hormone than sevelamer, calcium, lanthanum and placebo (Iron vs sevelamer -8.6 pg/ml [95% CrI, -17.60 to -0.45], [95% PrI, -18.36 to 0.03]; moderate quality evidence) (Table 3).

**Table 3:** Direct, indirect, and NMA estimates of parathyroid hormone with 95% credible intervals and GRADE assessments from each pairwise comparison within the phosphate-binder network.

Treatment Comparison		Direct estimate; MD (95% CrI)	Quality of evidence	Indirect estimate; MD (95% CrI)	Quality of evidence	NMA estimate; MD (95% CrI)	Quality of evidence
Sevelamer	Calcium	12 (-6.89 to 31)	Low	15 (-131 to 163)	Low	13 (-5.18 to 30)	Low
Placebo	Sevelamer	66 (4 to 129)	High	6.65 (-73 to 84)	Very Low	26 (0.71 to 53)	Moderate^1^
Lanthanum	Sevelamer	54 (-18 to 127)	High	29.74 (-44 to 101)	High	66 (39 to 94)	High
Iron	Sevelamer	-8.7 (-17 to -0.26)	Very low	-13 (-135 to 105)	High	-8.6(-17 to -0.2)	Moderate^1^
Diet	Sevelamer	11 (-23 to 47)	High	Closed loop formed by a multi-arm trial	Not available	-5.4 (-37 to 26)	High
Calmag	Sevelamer	59 (1.7 to 116)	Moderate	No closed loop	Not available	58 (2.8 to 115)	Moderate
Placebo	Calcium	67 (3.6 to 131)	Moderate	-14 (-64 to 92)	Low	13 (-12 to 41)	Low^1^
Lanthanum	Calcium	44 (9.2 to 80)	Low	76 (-47 to 200)	Low	53 (26 to 81)	Very Low^1^
Diet	Calcium	-26 (-59 to 6.8)	High	-159 (-348 to 26)	Low	-18 (-49 to 13)	High
Lanthanum	Placebo	40 (29 to 50)	High	-5 (-148 to 136)	Low	39 (28 to 50)	Moderate^1^
Iron	Placebo	-30 (-68 to 6.3)	High	-36 (-141 to 70)	Very Low	-35 (-62 to -9.3)	High
Calsev	Iron	-20 (-26 to -15)	High	No closed loop	Not available	-20 (-27 to -14)	High
Iron	Calcium	NA	NA	-21 (-40 to -1.8)	Very Low	-21 (-40 to -1.8)	Very Low
Iron	Lanthanum	NA	NA	-75 (-102 to -50)	Very Low	-75 (-102 to -50)	Very Low
Diet	Placebo	NA	NA	-32 (-72 to 7)	Moderate	-32 (-72 to 7)	Moderate
Diet	Lanthanum	NA	NA	-71 (-111 to -31)	High	-71 (-111 to -31)	High
Diet	Iron	NA	NA	3.2 (-29 to 35)	Very Low	3.2 (-29 to 35)	Very Low
Calsev	Sevelamer	NA	NA	-29 (-40 to -19)	Very Low	-29 (-40 to -19)	Very Low
Calsev	Calcium	NA	NA	-42 (-62 to -21)	Very Low	-42 (-62 to -21)	Very Low
Calsev	Placebo	NA	NA	-56 (-84 to -29)	High	-56 (-84 to -29)	High
Calsev	Lanthanum	NA	NA	-95 (-124 to -68)	High	-95 (-124 to -68)	High
Calsev	Diet	NA	NA	-24 (-57 to 9.3)	Very Low	-24 (-57 to 9.3)	Very Low
Calmag	Calcium	NA	NA	45 (-13 to 105)	Low	45 (-13 to 105)	Low
Calmag	Placebo	NA	NA	31 (-29 to 94)	Moderate	31 (-29 to 94)	Moderate
Calmag	Lanthanum	NA	NA	-8.2 (-69 to 55)	Moderate	-8.2 (-69 to 55)	Moderate
Calmag	Iron	NA	NA	66 (11 to 124)	Very Low	66 (11 to 124)	Very Low
Calmag	Diet	NA	NA	63 (-0.3 to 128)	Moderate	63 (-0.3 to 128)	Moderate
Calmag	Calsev	NA	NA	87 (31 to 145)	Very Low	87 (31 to 145)	Very Low

Note:

^1^Rated down for incoherence.

Abbreviations: CrI: Credible interval; MD: Mean difference; calmag: calcium and magnesium; calsev: calcium and sevelamer; calsevlant: calcium or sevelamer or lanthanum.

Combination treatment with magnesium yielded significantly higher parathyroid hormone levels than iron and calcium-and-sevelamer combination. Combination treatment with sevelamer and calcium showed lower parathyroid hormone levels as compared to single treatment with sevelamer, calcium, lanthanum and iron.

Of the 28 comparisons, 11 failed to reach statistical significance in the network estimate. We classified eight as high quality, eight as moderate quality, three as low quality evidence and nine as very low quality (Table 3).

**Fig 9:**
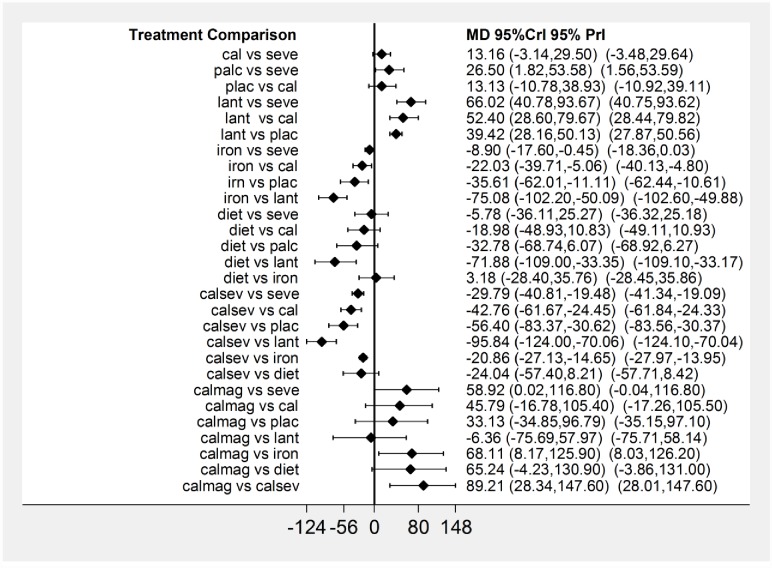
Network meta-analysis results for serum parathyroid hormone. Forest plot of effectiveness outcome for mean parathyroid hormone reduction at the end of the study period; MD: Mean difference; Crl: Credible interval; PrI: predictive interval.

Patients treated with sevelamer and calcium combination had a higher likelihood of reduction in serum parathyroid hormone as compared to those treated with other treatment categories (median SUCRA, 1; 95% CrI, 0.86 to 1) (Fig 5) (Table N in the S1 Supporting Information File). The between-study variance was 1.29 (95% CrI, 0.00 to 12.00) and considered as high heterogeneity. Fig 9 shows the rank-heat plot of the phosphate binder network for laboratory outcomes.

### Assessment of robustness of our findings

In meta-regression analysis, trial duration is not associated with a significant change in phosphate, calcium and parathyroid hormone levels (regression coefficient for phosphate, 0.009 [95% CrI, -0.019 to 0.038]; regression coefficient for calcium, 0.011 [95% CrI, -0.005 to 0.027]; regression coefficient for parathyroid hormone, -0.186 [95% CrI, -1.847 to 1.338]) (Table O in the S1 Supporting Information File).

We performed subgroup analysis by comparing studies with and without dialysis patient populations. The following three treatment comparisons were employed in both dialysis and non-dialysis groups for the phosphate outcome: Calcium vs Sevelamer, placebo vs. Sevelamer and placebo vs. calcium (MD = 0.03 [95% CrI, -0.76 to 0.76 in the dialysis group; MD = 0.19 [95% CrI, -0.34 to 0.72] for non-dialysis group for calcium vs. Sevelamer; MD = -0.44 [95% CrI, -1.61 to 0.57] in the dialysis group; MD = 1.09 [95% CrI, 0.06 to 2.11] in the non-dialysis group for placebo vs. Sevelamer; MD = -0.46 [95% CrI, -1.85 to 0.79] in the dialysis group, MD = 0.90 [95% CrI, -0.10 to 1.91] in the non-dialysis group for placebo vs. Sevelamer).

No evidence of inconsistency between direct and indirect estimates were found for all three outcomes (p = 0.62 for phosphate, p = 0.70 for calcium and p = 0.38 for parathyroid hormone) (Table P in the S1 Supporting Information File).

## Discussion

### Summary of main findings

This NMA of 8397 participants from twenty-nine trials provide evidence for effectiveness of phosphate binders on laboratory outcomes in patients with CKD using both placebo-controlled and active-controlled trials. Our results indicate that all treatments likely result in reductions of serum phosphate relative to placebo (moderate to very low quality of evidence). Our NMA results find no statistically significant difference between active treatment categories in lowering serum phosphate. Further, combination therapy provides no benefits relative to monotherapy in lowering serum phosphate.

In terms of reducing serum calcium levels, we find no statistically significant difference between those who receive placebo or active treatment. Calcium binders likely increase serum calcium levels relative to other interventions (moderate quality evidence for lanthanum and diet; low quality evidence for sevelamer).

The use of lanthanum increases parathyroid hormone as compared to sevelamer, calcium, iron, diet and placebo (high to very low quality of evidence). Our results show combination therapy with sevelamer and calcium will likely reduce parathyroid hormone as compared to single drug regimen which includes sevelamer, calcium, lanthanum or iron. Combination therapy with magnesium relative to iron and calcium-and-sevelamer combination yields an increase in parathyroid hormone levels with very low quality of evidence (66 pg/ml [95% CrI 11 to 124] for iron and 87 pg/ml [95% CrI, 31 to 145] for calcium-and-sevelamer).

We found no statistically significant association between trial duration and treatment effect in our meta-regression. However, this negative finding may be due to low power to detect an important effect. Hence, we cannot conclude that trial duration was not related to the effect size.

### Strengths and limitations of this study

This is the first network meta-analysis within a Bayesian framework that examined effectiveness of phosphate binders on laboratory outcomes in patients with CKD. The most recent systematic review addressing phosphate binders in patients with CKD did not report the effectiveness of calcium and NCBPBs on laboratory outcomes [21].

Strengths of our systematic review and meta-analysis include explicit eligibility criteria, a comprehensive search, independent duplicate assessment of eligibility, and use of the GRADE approach to assess quality of evidence an outcome-by-outcome basis for direct, indirect and network evidence. Limitations of our review included low and very low quality evidence for some treatment comparisons.

### Current knowledge and prior recommendations

Previous evidence had demonstrated that phosphate binders lower serum phosphate levels by diminishing phosphate reabsorption from the gastrointestinal system. Clinical practice guidelines recommend calcium as first line treatment for stages 4 and 5 CKD patients [9, 10] and suggest that serious gastrointestinal side effects, hypercalcemia and low parathyroid hormone at the lowest extreme (<100 pg/ml for hemodialysis patients) are main indications for a switch to NCBPs or a combination treatment [48, 74]. These recommendations ignore evidence that sevelamer results in decreased mortality relative to calcium [21, 75].

The association between laboratory outcomes and patient-important outcomes has been an area of interest for many researchers. A recent systematic review failed to show a significant association between drug effects on the laboratory outcomes and survival in CKD-MBD [23]. The trials included in this systematic review had low event rates in mortality due to inadequate trial duration and had flaws in the design and execution. Nevertheless, clinical practice guidelines still make recommendations for laboratory outcomes in the management of CKD-MBD [8, 25–28].

Comparative effectiveness of phosphate binders on markers of bone and mineral metabolism including phosphate, calcium and parathyroid hormone have been investigated *in vivo* and *in vitro* studies. An association between calcium phosphate binders and cardiovascular calcifications, positive calcium balance and hypercalcemia have been previously reported [16, 17, 76].

In animal models, iron has been associated with a significant decline in serum parathyroid levels [77]. In contrary to those findings, iron administration has been linked to an increase in parathyroid hormone levels in a small-scale observational study over a 12-week follow-up in dialysis patients [78].

Magnesium has been inversely correlated with parathyroid hormone and plays a role in the causation of adynamic bone disorder [79–81]. Therefore, magnesium is not recommended as first line treatment for hyperphosphatemia. However, our review with one trial and 252 participants did not find statistical evidence of a change in parathyroid hormone with magnesium intake.

### Implications of the review for mechanisms

Sevelamer reduces mortality relative to calcium [22], but results did not indicate a superior effect in lowering phosphate or parathyroid hormone. This review also supported previous findings related to the link between the use of calcium and hypercalcemia. Therefore, the only link between laboratory values and mortality reduction may be serum calcium levels.

### Implications of the review for research

According to our previous systematic review, the effects of various types of NCBPs have not been linked to mortality, although the sevelamer and calcium comparison yielded significant results supporting the mortality benefit of sevelamer [22]. Further research is needed with adequate trial duration and size to address the relative impact of phosphate binders on mortality and other patient-important outcomes.

Some RCTs are designed and executed to assess the impacts of treatments on laboratory outcomes rather than mortality or quality of life. They often report events during the trial period, but study durations are not long enough to capture the effects on patient important outcomes. Since it is not always possible and practical to design and conduct an RCT to capture information about patient important outcomes, laboratory outcomes are used instead. The main problem with this approach is that it is difficult to relate laboratory outcomes to patient-important outcomes.

## Conclusion and future directions

This NMA showed only small and unconvincing differences between phosphate binding agents with low to very low quality of evidence. The treatment of hyperphosphatemia with calcium will likely induce hypercalcemia. The combination therapy with sevelamer and calcium will likely cause a decrease in serum parathyroid hormone.

The only result possibly explaining the previously demonstrated reduction in mortality with sevelamer versus calcium binders was a lower serum calcium with sevelamer. Our findings emphasize the necessity for trials focusing on patient-important outcomes to establish the relative benefit and harm of alternative management strategies for CKD-MBD.

In order to fully explore the return on investment and risk of investment, cost and effectiveness data should be incorporated in a network meta-analyses. This will guide policy-makers in drug coverage making decisions, especially in countries with taxed-based health care financing systems, such as Canada.

## Supporting information

S1 Supporting Information File File A. The PRISMA NMA checklist. The PRISMA NMA checklist. File B. Search strategies. Search strategies for MEDLINE OVID, EMBASE OVID and EBM Reviews—Cochrane Central Register of Controlled Trials databases. Fig A. Assessment of publication bias by funnel plots for phosphate outcome. Funnel plot of effectiveness outcome for mean phosphate reduction at the end of the study period. Table A. Study Characteristics. Table B. Treatment codes, treatment categories and abbreviations used in the analysis. Table C. Treatment comparisons, number of studies and number of patients for phosphate outcome. Table D. GRADE quality assessment of direct evidence from each pairwise treatment comparison for phosphate. Table E. GRADE confidence assessments of indirect estimates per pairwise treatment comparison for phosphate in cases when direct comparisons are available. Table F. GRADE confidence assessments of indirect estimates per pairwise treatment comparison for phosphate in cases when direct comparisons are unavailable. Table G. GRADE quality assessment of direct evidence from each pairwise treatment comparison for calcium. Table H. GRADE confidence assessments of indirect estimates per pairwise treatment comparison for calcium when direct comparisons are available. Table I. GRADE confidence assessments of indirect estimates per pairwise treatment comparison for calcium when direct comparisons are unavailable. Table K. GRADE quality assessment of direct evidence from each pairwise treatment comparison for parathyroid hormone. Table L. GRADE confidence assessments of indirect estimates per pairwise treatment comparison for parathyroid hormone when direct comparisons are available. Table M. GRADE confidence assessments of indirect estimates per pairwise treatment comparison for parathyroid hormone when direct comparisons are unavailable. Table N. SUCRA rankings of phosphate binders. Table O. Effectiveness outcome for mean phosphate, calcium and parathyroid hormone reductions at the end of the study period using adjusted analysis for trial duration. Table P. Exploring the global inconsistency in networks for each laboratory outcome using the design-by-treatment interaction model.
(DOCX)
