# Supplementary material for: Effects of different phosphate lowering strategies in patients with CKD on laboratory outcomes: A systematic review and NMA
Source: PLoS One. 2017 Mar 1;12(3):e0171028. doi: 10.1371/journal.pone.0171028 (PMC5331957; doi:10.1371/journal.pone.0171028)
Supplement: S1 Supporting Information File — File A. The PRISMA NMA checklist. The PRISMA NMA checklist. File B. Search strategies. Search strategies for MEDLINE OVID, EMBASE OVID and EBM Reviews—Cochrane Central Register of Controlled Trials databases. Fig A. Assessment of publication bias by funnel plots for phosphate outcome. Funnel plot of effectiveness outcome for mean phosphate reduction at the end of the study period. Table A. Study Characteristics. Table B. Treatment codes, treatment categories and abbreviations used in the analysis. Table C. Treatment comparisons, number of studies and number of patients for phosphate outcome. Table D. GRADE quality assessment of direct evidence from each pairwise treatment comparison for phosphate. Table E. GRADE confidence assessments of indirect estimates per pairwise treatment comparison for phosphate in cases when direct comparisons are available. Table F. GRADE confidence assessments of indirect estimates per pairwise treatment comparison for phosphate in cases when direct comparisons are unavailable. Table G. GRADE quality assessment of direct evidence from each pairwise treatment comparison for calcium. Table H. GRADE confidence assessments of indirect estimates per pairwise treatment comparison for calcium when direct comparisons are available. Table I. GRADE confidence assessments of indirect estimates per pairwise treatment comparison for calcium when direct comparisons are unavailable. Table K. GRADE quality assessment of direct evidence from each pairwise treatment comparison for parathyroid hormone. Table L. GRADE confidence assessments of indirect estimates per pairwise treatment comparison for parathyroid hormone when direct comparisons are available. Table M. GRADE confidence assessments of indirect estimates per pairwise treatment comparison for parathyroid hormone when direct comparisons are unavailable. Table N. SUCRA rankings of phosphate binders. Table O. Effectiveness outcome for mean phosphate, calcium and parathyroid hormone reductions at the [file pone.0171028.s001.docx]

**S1 Supporting Information File**

Comparative effectiveness of phosphate binders in patients with chronic kidney disease: A systematic review and network meta-analysis. Nigar Sekercioglu, Areti Angeliki Veroniki, Lehana Thabane, Jason W. Busse, Noori Akhtar-Danesh, Alfonso Iorio, Luciane Cruz Lopes, Gordon H. Guyatt.

**Data Availability:** All relevant data are within the paper and its Supporting Information files.

**File A. The PRISMA NMA checklist.** The PRISMA NMA checklist.

**File B. Search strategies.** Search strategies for MEDLINE OVID, EMBASE OVID and EBM Reviews - Cochrane Central Register of Controlled Trials databases.

**Fig A.** **Assessment of publication bias by funnel plots for phosphate outcome.** Funnel plot of effectiveness outcome for mean phosphate reduction at the end of the study period.

**Table A. Study Characteristics.**

**Table B.** **Treatment codes, treatment categories and abbreviations used in the analysis.**

**Table C. Treatment comparisons, number of studies and number of patients for phosphate outcome.**

**Table D.** **GRADE quality assessment of direct evidence from each pairwise treatment comparison for phosphate.** For domains “Study Limitations”, “Precision”, “Consistency”, and “Directness”: No serious limitations, Serious limitations or Very serious limitations. For the domain “Publication bias”: Not likely, Likely to exist or not applicable if the comparison has less than ten trials. Reasons are provided when rating down. All direct comparisons begin with a “High” rating.^1^Rated down two levels for imprecision;^2^We employed random effect models, ^3^Bayesian methods used, ^4^The frequentist method used. CI: Confidence interval; Crl: credible intervals; MD: mean difference; N/A: not applicable. I^2^ indicates the expected degree of change in the effect estimates due to between-study variance.

**Table E.** **GRADE confidence assessments of indirect estimates per pairwise treatment comparison for phosphate in cases when direct comparisons are available.** A single first order loop for each pairwise comparison is used to GRADE indirect estimates. All indirect comparisons begin with the lower of the two contributing direct estimates and undergo an assessment of transitivity.; The quality of evidence rating for the indirect comparisons informing each paired comparison was the lower of the ratings of quality for the direct estimates contributing to the first or higher order loop. Abbreviations: calmag: calcium and magnesium; calsev: calcium and sevelamer; calsevlant: calcium or sevelamer or lanthanum; H: high; Low: low; M: moderate; VL: very low.

**Table F. GRADE confidence assessments of indirect estimates per pairwise treatment comparison for phosphate in cases when direct comparisons are unavailable**. A single first order loop for each pairwise comparison is used to GRADE indirect estimates. All indirect comparisons begin with the lower of the two contributing direct estimates and undergo an assessment of transitivity.; The quality of evidence rating for the indirect comparisons informing each paired comparison was the lower of the ratings of quality for the direct estimates contributing to the first or higher order loop. Abbreviations: calmag: calcium and magnesium; calsev: calcium and sevelamer; calsevlant: calcium or sevelamer or lanthanum; H: high; Low: low; M: moderate; VL: very low.

**Table G. GRADE quality assessment of direct evidence from each pairwise treatment comparison for calcium.** For domains “Study Limitations”, “Precision”, “Consistency”, and “Directness”: No serious limitations, Serious limitations or Very serious limitations. For the domain “Publication bias”: Not likely, Likely to exist or not applicable if the comparison has less than ten trials. Reasons are provided when rating down. All direct comparisons begin with a “High” rating.^1^Rated down two levels for imprecision;^2^We employed random effect models, ^3^Bayesian methods used, ^4^The frequentist method used. CI: Confidence interval; Crl: credible intervals; MD: mean difference; N/A: not applicable. I^2^ indicates the expected degree of change in the effect estimates due to between-study variance.

**Table H. GRADE confidence assessments of indirect estimates per pairwise treatment comparison for calcium when direct comparisons are available.** A single first order loop for each pairwise comparison is used to GRADE indirect estimates. All indirect comparisons begin with the lower of the two contributing direct estimates and undergo an assessment of transitivity.; The quality of evidence rating for the indirect comparisons informing each paired comparison was the lower of the ratings of quality for the direct estimates contributing to the first or higher order loop. Abbreviations: calmag: calcium and magnesium; calsev: calcium and sevelamer; calsevlant: calcium or sevelamer or lanthanum; H: high; Low: low; M: moderate; VL: very low.

**Table I. GRADE confidence assessments of indirect estimates per pairwise treatment comparison for calcium when direct comparisons are unavailable.** A single first order loop for each pairwise comparison is used to GRADE indirect estimates. All indirect comparisons begin with the lower of the two contributing direct estimates and undergo an assessment of transitivity.; The quality of evidence rating for the indirect comparisons informing each paired comparison was the lower of the ratings of quality for the direct estimates contributing to the first or higher order loop. Abbreviations: calmag: calcium and magnesium; calsev: calcium and sevelamer; calsevlant: calcium or sevelamer or lanthanum; H: high; Low: low; M: moderate; VL: very low.

**Table K. GRADE quality assessment of direct evidence from each pairwise treatment comparison for parathyroid hormone.** For domains “Study Limitations”, “Precision”, “Consistency”, and “Directness”: No serious limitations, Serious limitations or Very serious limitations. For the domain “Publication bias”: Not likely, Likely to exist or not applicable if the comparison has less than ten trials. Reasons are provided when rating down. All direct comparisons begin with a “High” rating.^1^Rated down two levels for imprecision;^2^We employed random effect models, ^3^Bayesian methods used, ^4^The frequentist method used. CI: Confidence interval; Crl: credible intervals; MD: mean difference; N/A: not applicable. I^2^ indicates the expected degree of change in the effect estimates due to between-study variance.

**Table L. GRADE confidence assessments of indirect estimates per pairwise treatment comparison for parathyroid hormone when direct comparisons are available.** A single first order loop for each pairwise comparison is used to GRADE indirect estimates. All indirect comparisons begin with the lower of the two contributing direct estimates and undergo an assessment of transitivity.; The quality of evidence rating for the indirect comparisons informing each paired comparison was the lower of the ratings of quality for the direct estimates contributing to the first or higher order loop. Abbreviations: calmag: calcium and magnesium; calsev: calcium and sevelamer; calsevlant: calcium or sevelamer or lanthanum; H: high; Low: low; M: moderate; VL: very low.

**Table M. GRADE confidence assessments of indirect estimates per pairwise treatment comparison for parathyroid hormone when direct comparisons are unavailable.** A single first order loop for each pairwise comparison is used to GRADE indirect estimates. All indirect comparisons begin with the lower of the two contributing direct estimates and undergo an assessment of transitivity.; The quality of evidence rating for the indirect comparisons informing each paired comparison was the lower of the ratings of quality for the direct estimates contributing to the first or higher order loop. Abbreviations: calmag: calcium and magnesium; calsev: calcium and sevelamer; calsevlant: calcium or sevelamer or lanthanum; H: high; Low: low; M: moderate; VL: very low.

**Table N. SUCRA rankings of phosphate binders.** The results of surface under the cumulative ranking curve of eight phoshate binders; CrI: Credible interval; MD: Mean difference; SUCRA: surface under the cumulative ranking curve.

**Table O. Effectiveness outcome for mean phosphate, calcium and parathyroid hormone reductions at the end of the study period using adjusted analysis for trial duration.** There is no significant association between treatment effect and trial duration as credible intervals include zero; MD: Mean difference; CrI: Credible interval.

**Table P. Exploring the global inconsistency in networks for each labarotary outcome using the design-by-treatment interaction model.**

**File B** Search strategies MEDLINE OVID, EMBASE OVID

| (((kidney* or nephro* or renal or home or peritoneal or intermittent or chronic or extracorporeal or ambulatory) adj2 (haemodialys* or hemodialys* or dialys*)) or hemorenodialysis or hemodialyse or CAPD).ti,ab. |
| --- |
| renal dialysis/ or hemodialysis, home/ or peritoneal dialysis/ or peritoneal dialysis, continuous ambulatory/ |
| renal insufficiency, chronic/ or kidney failure, chronic/ |
| (((chronic or "end-stage" or "end stage") adj3 (kidney* or renal or nephro*) adj3 (insufficien* or disease*)) or esrd).ti,ab. |
| renal osteodystrophy/ or ((renal or kidney* or nephro*) adj2 (osteodystroph* or ricket*)).mp. |
| azotemia/ or azotemi*.mp |
| uremia/ or uremi*.mp. |
| 1 or 2 or 3 or 4 or 5 or 6 or 7 |
| controlled clinical trial.pt. or controlled clinical trials as topic/ or meta analysis.pt. or meta analysis as topic/ or multicentre study.pt. or multicenter studies as topic/ or randomized controlled trial.pt. or randomized controlled trials as topic/ or pragmatic clinical trial.pt. or Pragmatic Clinical Trials as Topic/ or ((preference or practical or pragmatic or "real world" or naturalistic) adj5 trial*).ti,ab. or Comparative Effectiveness Research/ or ((comparative adj2 effectiveness) or (CER adj5 (research* or method* or framework* or compari* or statement*))).ti,ab. or ((singl: or doubl: or tripl: or trebl:) and (mask: or blind:)).ti,ab. or ((random: adj5 trial:) or rct or rcts).ti,ab. |
| calcium/ or (calc* or calc* acet* or Calc* acet* or Cal* car* or Cal*Car).mp. [mp=title, abstract, original title, name of substance word, subject heading word, keyword heading word, protocol supplementary concept word, rare disease supplementary concept word, unique identifier] |
| (phosphate binders or phosphate lowering agent).mp. [mp=title, abstract, original title, name of substance word, subject heading word, keyword heading word, protocol supplementary concept word, rare disease supplementary concept word, unique identifier] |
| (lanthanum or lanthanum carbonate).mp. [mp=title, abstract, original title, name of substance word, subject heading word, keyword heading word, protocol supplementary concept word, rare disease supplementary concept word, unique identifier] |
| (sevelamer or sevela*).mp. [mp=title, abstract, original title, name of substance word, subject heading word, keyword heading word, protocol supplementary concept word, rare disease supplementary concept word, unique identifier] |
| (iron or ferrous citrate).mp. [mp=title, abstract, original title, name of substance word, subject heading word, keyword heading word, protocol supplementary concept word, rare disease supplementary concept word, unique identifier] |
| 10 or 11 or 12 or 13 or 14 |
| 8 and 9 and 14 |
| limit 25 to yr="2013 -Current" |

EBM Reviews - Cochrane Central Register of Controlled Trials

| 1 | chronic kidney disease:ti, ab, kw | 4911 |
| --- | --- | --- |
| 2 | Phosphate binders | 266 |
| 3 | randomized controlled trials | 571289 |
| 4 | 1 and 2 and 3 | 82 |

**Figure A.** Assessment of publication bias by funnel plots for phosphate outcome


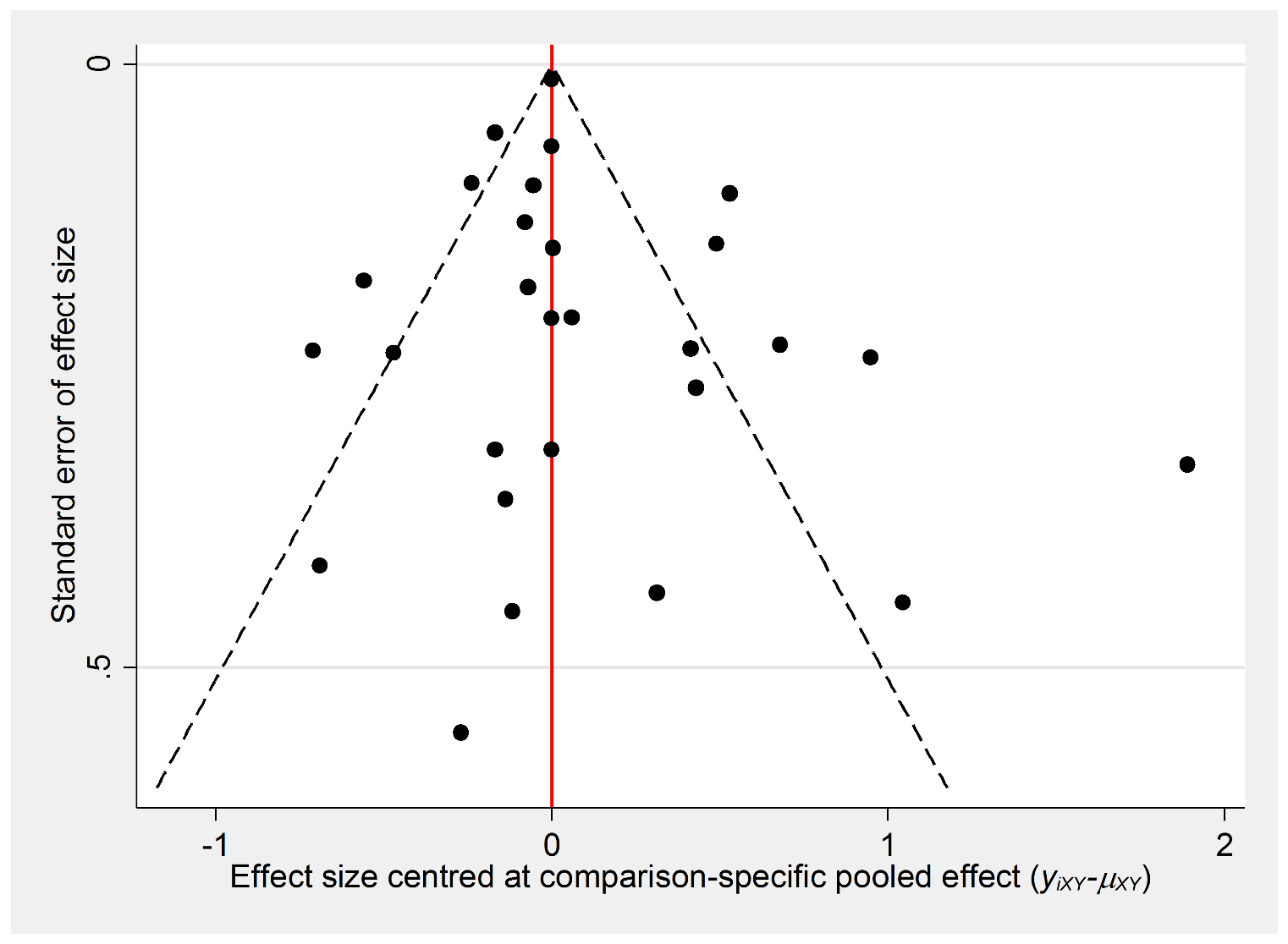


Note: Funnel plot of effectiveness outcome for mean phosphate reduction at the end of the study period

**Table A.** Study Characteristics

| Study, Year (Reference) | Country | Randomly assigned patients, n | Number of arms | Women, % | Age, y (SD) | Stage of CKD | Comparison | Follow-up duration in months |
| --- | --- | --- | --- | --- | --- | --- | --- | --- |
| Chertow et al, 2002[15] | United States, Austria and Germany | 99  101 | 2 | 70, 35% | 57 (14)  56 (16) | Stage 5D | Sevelamer vs. calcium | 12 |
| Sadek et al, 2003[16] | France | 21  21 | 2 | - | - | Stage 5D | Sevelamer vs. calcium | 5 |
| Block et al, 2007 [17] | United States and Italy | 60  67 | 2 | 42%  36% | 56 (14)  58 (14) | Stage 5D | Sevelamer vs. calcium | 60 |
| Russo et al, 2007 [18] | Italy | 30  30  30 | 3 | 3 (10%)  5 (16%) | 55 (13)  54 (12) | Non-dialysis | Sevelamer vs. calcium vs. phosphorus restricted diet | 24 |
| Barreto et al, 2008 [19] | Brazil | 52  49 | 2 | 34%  30% | 47 (13)  47 (14) | Stage 5D | Sevelamer vs. calcium | 12 |
| Qunibi et al, 2008 [20] | United States | 100  103 | 2 | 54%  42% | 60 (12)  58 (12) | Stage 5D | Sevelamer vs. calcium | 12 |
| Suki et al, 2008[21] | United States | 1053  1050 | 2 | 479 (45%)  481(48%) | 59 (14)  60 (15) | Stage 5D | Sevelamer vs. calcium | 45 |
| Takei et al, 2008[22] | Japan | 22  20 | 2 | 50%  45% | 54 (10)  54 (9) | Stage 5D | Sevelamer vs. calcium | 6 |
| Wilson et al, 2009 [23] | United States and United Kingdom | 680  674 | 2 | 42%  38% | 54 (14)  60 (14) | Stage 5D | Lanthanum vs.  Standard treatment | 24 |
| De Francisco et al, 2010[24] | Spain, Portugal, Germany, Italy, Romania and Poland | 127  125 | 2 | 49%  47% | 56 (12)  59 (14) | Stage 5D | Sevelamer vs. calcium | 6 |
| Gulati et al,  2010[25] | India | 11  11 | 2 | 50%  45% | 10 (5)  10(5) | Non-dialysis | Sevelamer vs. calcium | 3 |
| Kakuta et al, 2011 [26] | Japan | 91  92 | 2 | 43%  49% | 59 (12)  57 (12) | Stage 5D | Sevelamer vs. calcium | 12 |
| Toussaint et al, 2011 [27] | Australia | 22  23 | 2 | 45%  26% | 56 (15)  59 (15) | Stage 5D | Lanthanum vs calcium | 18 |
| Block et al, 2012[28] | United States, Germany and United Kingdom | 57  28  30  30 | 4 | 21%  18%  20%  20% | 65 (12)  70 (10)  66 (12)  68 (12) | Non-dialysis | Placebo vs.  Lanthanum vs.  Sevelamer vs.  Calcium | 10 (median follow-up time 249 days) |
| Di Iorio et al, 2012[29] | United States and Italy | 232  234 | 2 | 50%  52% | 67 (14)  65 (15) | Stage 5D | Sevelamer vs. calcium | 24 |
| Di Iorio et al, 2013 [30] | Italy | 121  118 | 2 | 39%  39% | 57 (12)  59 (12) | Non-dialysis | Sevelamer vs. calcium | 36 |
| Lee et al, 2013 [31] | Korea | 50 | 2 | 45%  63% | 48 (11)  52 (11) | Stage 5D | Lanthanum vs calcium | 6 |
| Ohtake et al, 2013 [32] | Japan | 26  26 | 2 | 40% | 68 (6) | Stage 5D | Lanthanum vs calcium | 12 |
| Wuthrich et al, 2013 [33] | Canada, United States, Romania, and Switzerland | 24  126 | 2 | 58%  37% | 60 (13)  62 (11) | Stage 5D | Sevelamer vs. sucroferric oxyhydroxide | 1.5 |
| Xu et al, 2013 [34] | China | 115  115 | 2 | 47%  36% | 48 (13)  48 (12) | Stage 5D | Lanthanum vs. Placebo | 2 |
| Floege et al, 2014[35] | United States, Romania, Germany and Switzerland | 349  710 | 2 | 37%  45% | 56 (15)  56 (13) | Stage 5D | Sevelamer vs. sucroferric oxyhydroxide | 6 |
| Takahara et al, 2014 [36] | Japan | 86  55 | 2 | 55%  27% | 61 (11)  62 (13) | Non-dialysis | Lanthanum vs. Placebo | 2 |
| Urena-Torres et al, 2014 [37] | France | 17  12 | 2 | 41%  58% | 66 (15)  69 (13) | Non-dialysis | Lanthanum vs. Placebo | 3 |
| Wada et al, 2014 [38] | Japan | 21  22 | 2 | 23%  21% | 66 (10)  66 (8) | Stage 5D | Lanthanum vs calcium | 12 |
| Yokoyama et al, 2014 [39] | Japan and Unites States | 110  115 | 2 | 35%  37% | 62 (10)  60 (11) | Stage 5D | Sevelamer vs JTT-751 | 3 |
| Yokoyama et al, 2014 [40] | Japan and Unites States | 60  30 | 2 | 42%  41% | 65 (10)  65 (14) | Non-dialysis and dialysis | Ferric citrate vs. Placebo | 3 |
| Block et al, 2015[41] | United States, Germany and Spain | 75  74 | 2 | 69%  62% | 66 (12)  64 (14) | Non-dialysis and dialysis | Ferric citrate vs. Placebo | 3 |
| Lee et al, 2015 [42] | Taiwan | 36  75  72 | 3 | 37%  43%  31% | 53 (12)  53 (11)  53 (12) | Stage 5D | Ferric citrate vs. Placebo | 2 |
| Lewis et al, 2015 [43] | United States | 292  149 | 2 | 37%  42% | 56 (45-63)  54 (45-63) | Stage 5D | Ferric citrate vs.  Active control (calcium acetate and sevelamer) | 12 |

**Table B.** Treatment codes, treatment categories and abbreviations used in the analysis

| Code | Treatment name | Abbreviation |
| --- | --- | --- |
| 1 | sevelamer | seve |
| 2 | calcium | cal |
| 3 | placebo | plac |
| 4 | lanthanum | lant |
| 5 | iron | iron |
| 6 | Low phosphorus diet | Diet |
| 7 | Calcium and sevelamer | calsev |
| 8 | Calcium and magnesium | calmag |
| 9 | Calcium or Sevelamer or lanthanum | calsevlant |

**Table C.** Treatment comparisons, number of studies and number of patients for phosphate outcome

| Treatment Comparison | Coded Treatment Comparison | Number of studies | Number of patients |
| --- | --- | --- | --- |
| Cal vs. sev | 2 vs 1 | 10 | 3560 |
| Iron vs. sev | 5 vs 1 | 3 | 1336 |
| Diet vs. sev | 6 vs 1 | 2 | 120 |
| Calmag vs. sev | 8 vs 1 | 1 | 252 |
| Lant vs. cal | 4 vs 2 | 3 | 140 |
| Diet vs. cal | 6 vs 2 | 1 | 60 |
| Lant vs. plac | 4 vs 3 | 3 | 408 |
| Iron vs. plac | 5 vs 3 | 3 | 418 |
| Calsevlant vs plac | 9 vs 3 | 1 | 145 |
| Calsev vs. iron | 7 vs 5 | 1 | 441 |

**Table D.** GRADE quality assessment of direct evidence from each pairwise treatment comparison for phosphate outcome

| Treatment comparison | Number of head-to-head trials; n | Study Limitations | Precision | Consistency | Directness | Publication bias | Overall quality of evidence | Direct estimate^2,3^; MD (95% CrI) | Direct estimate^2^,^4;^ MD (95% CI) |
| --- | --- | --- | --- | --- | --- | --- | --- | --- | --- |
| Sevelamer vs.  Calcium | 10;3560 | Not serious | Serious limitations | Serious limitations  (I^2^:84%) | Not serious | Not likely | Low | 0.05 (-0.36 to 0.46) | 0.09 (-0.17 to 0.34) |
| Sevelamer vs. Iron | 3; 1303 | Serious (due to allocation concealment) | Serious limitations | Serious limitations  (I^2^:65%) | Not serious | N/A | Very Low | -0.28 (-1.06 to 0.45) | -0.16 (-0.51 to 0.19) |
| Sevelamer vs. diet | 1; 60 | Not serious | Very serious limitations^1^ | Serious limitations  (I^2^:94%) | Not serious | N/A | Very Low | -0.20 (-1.12 to 0.71) | -0.21 (-1.57 to 1.16) |
| Sevelamer vs. calmag | 1; 252 | Not serious | Very serious limitations^1^ | N/A | Not serious | N/A | Low | -0.17 (-0.59 to 0.23) | -0.18 (-0.59 to 0.23) |
| Lanthanum vs. Calcium | 3; 140 | Serious (due to allocation concealment) | Serious limitations | Not serious limitations  (I^2^:0%) | Not serious | N/A | Low | 0.15 (-0.69 to 0.98) | 0.16 (-0.30 to0.63) |
| Calcium vs. diet | 1; 30 | Not serious | No serious limitations | N/A | Not serious | N/A | High | -0.79 (-1.42 to -0.17) | -0.80 (-1.43 to -0.17) |
| Lanthanum vs Placebo | 3; 408 | Not serious | No serious limitations | Serious limitations  (I^2^:92%) | Not serious | N/A | Moderate | -0.87 (-1.6 to -0.14) | -0.89 (-1.73 to -0.05) |
| Iron vs. placebo | 3; 418 | Not serious | No serious limitations | Serious limitations  (I^2^:95%) | Not serious | N/A | Moderate | -1.49 (-2.2 to -0.69) | -1.49 (-2.58 to -0.40) |
| Placebo vs. calsevlant | 1; 145 | Not serious | Serious limitations | N/A | Not serious | N/A | Moderate | -0.09 (-0.23 to 0.03) | -0.10 (-0.23 to 0.03) |
| Iron vs Calsev | 1; 441 | Not serious | Serious limitations | N/A | Not serious | N/A | Moderate | 0.01 (-0.003 to 0.04) | 0.02 (-0.001 to 0.04) |
| Common within network between-study variance (95% Crl) = 0.15 (0.34 to 0.78) | | | | | | | | |  |

**Note:** For domains “Study Limitations”, “Precision”, “Consistency”, and “Directness”: No serious limitations, Serious limitations or Very serious limitations. For the domain “Publication bias”: Not likely, Likely to exist or not applicable if the comparison has less than ten trials. Reasons are provided when rating down. All direct comparisons begin with a “High” rating.^1^Rated down two levels for imprecision;^2^We employed random effect models, ^3^Bayesian methods used, ^4^The frequentist method used. CI: Confidence interval; Crl: credible intervals; MD: mean difference; N/A: not applicable. I^2^ indicates the expected degree of change in the effect estimates due to between-study variance.

**Table E.** GRADE confidence assessments of indirect estimates per pairwise treatment comparison for phosphate in cases when direct comparisons are available

| Treatment comparisons | Is a first order loop available? | Common comparator treatment in dominant first order loop (in the absence of first order loop, possible comparisons in higher order loop) | GRADE of first contributing direct comparison: name of the contributors (quality of evidence) | GRADE of second contributing direct comparison (quality of evidence) | Final GRADE of indirect comparison |
| --- | --- | --- | --- | --- | --- |
| Sevelamer vs.  Calcium | Yes | Diet | Diet calcium  (H) | Diet sevelamer  (L) | Low |
| Sevelamer vs. Iron | No | Iron placebo (M)  Placebo lanthanum (M)  Lanthanum calcium (L)  Calcium Sevelamer (L) | Not available | Not available | Low |
| Sevelamer vs. diet | Closed loop formed by a multi-arm trial | Not available | Not available | Not available | Not available |
| Sevelamer vs. calmag | Unconnected comparison | Unconnected comparison | Not available | Not available | Not available |
| Lanthanum vs. Calcium | No | Calcium sevelamer (L)  Sevelamer iron (VL)  Iron placebo (M)  Placebo lanthanum (M) | Not available | Not available | Very Low |
| Calcium vs. diet | Yes | Sevelamer | Diet sevalamer  (VL) | Calcium sevalamer  (L) | Very Low |
| Lanthanum vs. Placebo | No | Placebo Iron (M)  Iron sevelamer (VL)  Sevelamer calcium (L)  Calcium lanthanum (L) | Not available | Not available | Very Low |
| Iron vs. placebo | No | Placebo lanthanum (M)  Lanthanum calcium (L)  Calcium Sevelamer (L)  Sevelamer iron (VL) | Not available | Not available | Very Low |
| Placebo vs. Calsevlant | Unconnected comparison | Unconnected comparison | Not available | Not available | Not available |
| Iron vs. Calsev | Unconnected comparison | Unconnected comparison | Not available | Not available | Not available |

**Note:** A single first order loop for each pairwise comparison is used to GRADE indirect estimates. All indirect comparisons begin with the lower of the two contributing direct estimates and undergo an assessment of transitivity.; The quality of evidence rating for the indirect comparisons informing each paired comparison was the lower of the ratings of quality for the direct estimates contributing to the first or higher order loop. Abbreviations: calmag: calcium and magnesium; calsev: calcium and sevelamer; calsevlant: calcium or sevelamer or lanthanum; H: high; Low: low; M: moderate; VL: very low.

**Table F.** GRADE confidence assessments of indirect estimates per pairwise treatment comparison for phosphate in cases when direct comparisons are unavailable

| Treatment comparisons | First order loop available | Common comparator treatment in the dominant first order loop (in the absence of the first order loop, possible comparisons in higher order loop) | GRADE of first contributing direct comparison: name of the contributors (quality of evidence) | GRADE of second contributing direct comparison | Final GRADE of Indirect Comparison |
| --- | --- | --- | --- | --- | --- |
| Sevelamer vs. placebo | Yes | Iron | Sevelamer iron  (VL) | Placebo iron  (M) | Very Low |
| Lanthanum vs. sevelamer | Yes | calcium | Calcium sevelamer  (L) | Calcium lanthanum  (L) | Low |
| Calcium vs. placebo | Yes | Lanthanum | Placebo lanthanum  (M) | Calcium lanthanum  (L) | Low |
| Iron vs. lanthanum | No | Lanthanum placebo (M)  Placebo iron (M)  Iron Sevelamer (VL)  Sevelamer calcium (L)  Calcium lanthanum (L) | Not available | Not available | Very Low |
| Iron vs. calcium | Yes | Iron | Iron sevelamer  (L) | Calcium sevelamer  (VL) | Very low |
| Diet vs. placebo | No | Placebo lanthanum (M)  Lanthanum calcium (L)  Calcium Sevelamer (L)  Sevelamer diet (VL) | Not available | Not available | Very Low |
| Diet vs. lanthanum | Yes | Calcium | Diet calcium  (H) | Lanthanum calcium  (L) | Low |
| Iron vs. diet | Yes | Sevelamer | Iron sevelamer  (VL) | Diet sevelamer  (VL) | Very Low |
| Cal vs. calsev | No | Calcium Sevelamer (L)  Sevelamer iron (VL)  Iron calsev (M) | Not available | Not available | Very Low |
| Calsev vs. sevelamer | Yes | Iron | Calsev iron  (M) | Iron sevelamer  (VL) | Very Low |
| Calsev vs. placebo | Yes | Iron | Calsev iron  (M) | Placebo iron  (M) | Moderate |
| Calsev vs. lanthanum | No | Lanthanum placebo (M)  Placebo iron (M)  Iron calsev (M) | Not available | Not available | Moderate |
| Calsev vs. diet | No | Diet Sevelamer (VL)  Sevelamer iron (VL)  Iron calsev (M) | Not available | Not available | Very Low |
| Calmag vs. calcium | Yes | Sevelamer | Calcium vs. Sevelamer  (L) | Calmag vs. Sevelamer  (L) | Low |
| Calmag vs. placebo | No | Placebo lanthanum (M)  Lanthanum calcium (L)  Calcium Sevelamer (L)  Sevelamer calmag (L) | Not available | Not available | Low |
| Calmag vs. lanthanum | No | Lanthanum calcium (L)  Calcium Sevelamer (L)  Sevelamer calmag (L) | Not available | Not available | Low |
| Calmag vs. iron | Yes | Sevelamer | Calmag vs. Sevelamer  (L) | Iron vs. Sevelamer  (VL) | Very Low |
| Calmag vs. diet | Yes | Sevelamer | Calmag vs. Sevelamer  (L) | Diet vs. Sevelamer  (VL) | Very Low |
| Calmag vs. calsev | No | Calmag Sevelamer (L)  Sevelamer iron (VL)  Iron calsev (M) | Not available | Not available | Very Low |
| Calsevlant vs. sevelamer | No | Sevelamer iron (VL)  Iron placebo (M)  Placebo calsevlant (M) | Not available | Not available | Very Low |
| Calsevlant vs. calcium | No | Calcium lanthanum (L)  Lanthanum placebo (M)  Placebo calsevlant (M) | Not available | Not available | Low |
| Calsevlant vs. lanthanum | Yes | Placebo | Placebo vs. lanthanum  (M) | Calsevlant vs. placebo  (M) | Moderate |
| Calsevlant vs. iron | Yes | Placebo | Placebo vs. iron  (M) | Calsevlant vs. placebo  (M) | Moderate |
| Calsevlant vs. diet | No | Diet Sevelamer (VL)  Sevelamer iron (VL)  Iron placebo (M)  Placebo calsevlant (M) | Not available | Not available | Very Low |
| Calsevlant vs. calsev | No | Calsev iron (M)  iron vs. placebo (M)  Placebo calsevlant (M) | Not available | Not available | Moderate |
| Calsevlant vs. calmag | No | Calmag Sevelamer (L)  Sevelamer iron (VL)  Iron placebo (M)  Placebo calsevlant (M) | Not available | Not available | Very Low |

**Note:** A single first order loop for each pairwise comparison is used to GRADE indirect estimates. All indirect comparisons begin with the lower of the two contributing direct estimates and undergo an assessment of transitivity.; The quality of evidence rating for the indirect comparisons informing each paired comparison was the lower of the ratings of quality for the direct estimates contributing to the first or higher order loop. Abbreviations: calmag: calcium and magnesium; calsev: calcium and sevelamer; calsevlant: calcium or sevelamer or lanthanum; H: high; Low: low; M: moderate; VL: very low.

**Table G.** GRADE quality assessment of direct evidence from each pairwise treatment comparison for calcium outcome

| Treatment comparison | Number of head-to-head trials; n | Study Limitations | Precision | Consistency | Directness | Publication bias | Overall quality of evidence | Direct estimate^2^; MD (95% CrI) | Direct estimate^2^,^4;^ MD (95% CI) |
| --- | --- | --- | --- | --- | --- | --- | --- | --- | --- |
| Sevelamer vs.  Calcium | 11;3620 | Not serious | Not serious | Serious limitations  (I^2^:95%) | Not serious | Not serious | Moderate | 0.30 (0.08 to 0.51) | 0.29 (0.04 to 0.55) |
| Sevelamer vs. placebo | 1; 60 | Not serious | Not serious | N/A | Not serious | N/A | High | 0.10 (-0.39 to 0.59) | 0.60 (0.46 to 0.74) |
| Sevelamer vs. Lanthanum | 1; 58 | Not serious | Serious limitations | N/A | Not serious | N/A | Moderate | -0.09 (-0.33 to 0.13) | -0.10 (-0.33 to 0.13) |
| Sevelamer vs. Iron | 2; 381 | Serious (due to allocation concealment) | Serious limitations | Serious limitations  (I^2^:91%) | Not serious | N/A | Very low | -0.15 (-0.64 to 0.34) | -0.16 (-0.73 to 0.41) |
| Sevelamer vs. diet | 1; 60 | Not serious | Not serious | N/A | Not serious | N/A | Moderate | -0.60 (-0.74 to -0.45) | -0.60 (-0.74 to -0.46) |
| Sevelamer vs. calcium and magnesium | 1; 252 | Not serious | Not serious | N/A | Not serious | N/A | High | -012 (-0.26 to 0.02) | -012 (-0.27 to 0.03) |
| Placebo vs. calcium | 2; 147 | Not serious | Serious limitations | Serious limitations  (I^2^:72%) | Not serious | N/A | Low | -0.01 (-0.5 to 0.5) | -0.02 (-0.41 to 0.37) |
| Lanthanum vs. Calcium | 5; 248 | Serious (due to allocation concealment) | No serious limitations | No serious limitations  (I^2^:0%) | Not serious | N/A | Moderate | -0.33 (-0.67 to 0.009) | -0.30 (-0.47 to -0.14) |
| Lanthanum vs Placebo | 3; 408 | Not serious | No serious limitations | No serious limitations  (I^2^:0%) | Not serious | N/A | High | 0.07 (-0.33 to 0.48) | 0.12 (-0.09 to 0.33) |
| Iron vs. placebo | 3; 418 | Not serious | No serious limitations | No serious limitations  (I^2^:0%) | Not serious | N/A | High | 0.22 (-0.18 to 0.62) | 0.24 (-0.11 to 0.37) |
| Iron vs Calsev | 1; 441 | Not serious | No serious limitations | N/A | Not serious | N/A | High | 0.15 (0.13 to 0.16) | 0.15 (0.14 to 0.16) |
| Common within network between-study variance 0.10 (0.05 to 0.22) | | | | | | | | |  |

**Note:** For domains “Study Limitations”, “Precision”, “Consistency”, and “Directness”: No serious limitations, Serious limitations or Very serious limitations. For the domain “Publication bias”: Not likely, Likely to exist or not applicable if the comparison has less than ten trials. Reasons are provided when rating down. All direct comparisons begin with a “High” rating.^1^Rated down two levels for imprecision;^2^We employed random effect models, ^3^Bayesian methods used, ^4^The frequentist method used. CI: Confidence interval; Crl: credible intervals; MD: mean difference; N/A: not applicable. I^2^ indicates the expected degree of change in the effect estimates due to between-study variance.

**Table H.** GRADE confidence assessments of indirect estimates per pairwise treatment comparison for calcium when direct comparisons are available

| Treatment comparisons | First order loop available | Common comparator treatment in the dominant first order loop (in the absence of the first order loop, possible comparisons in higher order loop) | GRADE of first contributing direct comparison; name of the contributors (quality of evidence) | GRADE of second contributing direct comparison | Final GRADE of Indirect Comparison |
| --- | --- | --- | --- | --- | --- |
| Sevelamer vs.  Calcium | Yes | Lanthanum | Calcium vs. lanthanum  (M) | Sevelamer vs. lanthanum  (M) | Moderate |
| Sevelamer vs. placebo | Yes | Calcium | Placebo calcium  (L) | Sevelamer vs. calcium  (M) | Low |
| Sevelamer vs. Lanthanum | Yes | Calcium | Sevelamer calcium  (M) | Calcium lanthanum  (M) | Moderate |
| Sevelamer vs. Iron | Yes | Placebo | Placebo Sevelamer  (H) | Placebo iron  (H) | High |
| Sevelamer vs. diet | Unconnected comparison | Not available | Not available | Not available | Not available |
| Sevelamer vs. calmag | Unconnected comparison | Not available | Not possible | Not possible | Not available |
| Placebo vs. calcium | Yes | Lanthanum | Lanthanum placebo  (H) | Calcium lanthanum  (M) | Moderate |
| Lanthanum vs. Calcium | Yes | Placebo | Lanthanum placebo  (H) | Calcium placebo  (L) | Low |
| Lanthanum vs. Placebo | Yes | Calcium | Calcium placebo  (L) | Calcium lanthanum  (M) | Low |
| Iron vs. placebo | Yes | Sevelamer | Iron sevelamer  (VL) | Placebo sevelamer  (H) | Very Low |
| Iron vs. Calsev | Unconnected comparison | Not available | Not possible | Not possible | Not available |

**Note:** A single first order loop for each pairwise comparison is used to GRADE indirect estimates. All indirect comparisons begin with the lower of the two contributing direct estimates and undergo an assessment of transitivity.; The quality of evidence rating for the indirect comparisons informing each paired comparison was the lower of the ratings of quality for the direct estimates contributing to the first or higher order loop. Abbreviations: calmag: calcium and magnesium; calsev: calcium and sevelamer; calsevlant: calcium or sevelamer or lanthanum; H: high; Low: low; M: moderate; VL: very low.

**Table I.** GRADE confidence assessments of indirect estimates per pairwise treatment comparison for calcium when direct comparisons are unavailable

| Treatment comparisons | First order loop available | Common comparator treatment in the dominant first order loop (in the absence of the first order loop, possible comparisons in higher order loop) | GRADE of first contributing direct comparison; name of the contributors (quality of evidence) | GRADE of second contributing direct comparison | Final GRADE of Indirect Comparison |
| --- | --- | --- | --- | --- | --- |
| Iron vs. calcium | Yes | Sevelamer | Iron sevelamer  (VL) | Calcium sevelamer  (M) | Very Low |
| Iron vs. lanthanum | Yes | Sevelamer | Iron sevelamer  (VL) | Lanthanum sevelamer  (M) | Very Low |
| Diet vs. calcium | Yes | Sevelamer | Diet sevelamer  (M) | Calcium sevelamer  (M) | Moderate |
| Diet vs. placebo | Yes | Sevelamer | Diet sevelamer  (M) | Placebo sevelamer  (H) | Moderate |
| Diet vs. lanthanum | Yes | Sevelamer | Diet sevelamer  (M) | Lanthanum sevelamer  (M) | Moderate |
| Diet vs. Iron | Yes | Sevelamer | Diet sevelamer  (M) | Iron sevelamer  (VL) | Very Low |
| Calsev vs. sevelamer | Yes | Iron | Sevelamer iron  (VL) | Iron calsev  (H) | Very Low |
| Calsev vs. calcium | No | Calcium Sevelamer (M)  Sevelamer iron (VL)  Iron calsev (H) | Not available | Not available | Very Low |
| Calsev vs. placebo | Yes | Iron | Placebo iron  (H) | Calsev iron  (H) | High |
| Calsev vs. lanthanum | No | Lanthanum calcium (M)  Calcium Sevelamer (M)  Sevelamer iron (VL)  Iron calsev (H) | Not available | Not available | Very Low |
| Calsev vs. diet | No | Diet Sevelamer (M)  Sevelamer iron (VL)  Iron calsev (H) | Not available | Not available | Very Low |
| Calmag vs. calcium | Yes | Sevelamer | Calcium sevelamer  (M) | Calmag sevelamer  (H) | Moderate |
| Calmag vs. placebo | Yes | Sevelamer | Placebo sevelamer  (H) | Calmag sevelamer  (H) | High |
| Calmag vs. lanthanum | Yes | Sevelamer | Lanthanum sevelamer  (M) | Calmag sevelamer  (H) | Moderate |
| Calmag vs. iron | Yes | Sevelamer | Sevelamer iron  (VL) | Calmag vs. Sevelamer  (H) | Very Low |
| Calmag vs. diet | Yes | Sevelamer | Diet sevelamer  (M) | Calmag vs. Sevelamer  (H) | Moderate |
| Calmag vs. calsev^a^ | No | Calmag Sevelamer (H)  Sevelamer iron (VL)  Iron calsev (H) | Not available | Not available | Very Low |

**Note:** A single first order loop for each pairwise comparison is used to GRADE indirect estimates. All indirect comparisons begin with the lower of the two contributing direct estimates and undergo an assessment of transitivity.; The quality of evidence rating for the indirect comparisons informing each paired comparison was the lower of the ratings of quality for the direct estimates contributing to the first or higher order loop. Abbreviations: calmag: calcium and magnesium; calsev: calcium and sevelamer; calsevlant: calcium or sevelamer or lanthanum; H: high; Low: low; M: moderate; VL: very low.

**Table K.** GRADE quality assessment of direct evidence from each pairwise treatment comparison for parathyroid hormone outcome

| Treatment comparison | Number of head-to-head trials; n | Study Limitations | Precision | Consistency | Directness | Publication bias | Overall quality of evidence | Direct estimate^2^; MD (95% CrI) | Direct estimate^2^,^4;^ MD (95% CI) |
| --- | --- | --- | --- | --- | --- | --- | --- | --- | --- |
| Sevelamer vs. calcium | 11; 3620 | Not serious | Serious limitations | Serious limitations; (I^2^: 58%) | No serious limitations | No serious limitations | Low | 12 (-6.89 to 31) | 4.63 (-31 to 41) |
| Placebo vs. sevelamer | 1; 60 | Not serious | No serious limitations | N/A | No serious limitations | N/A | High | 66 (4 to 129) | 172 (20 to 323) |
| Lanthanum vs. sevelamer | 1; 58 | Not serious | Serious limitations | N/A | No serious limitations | N/A | High | 54 (-18 to 127) | 48 (-31 to 127) |
| Iron vs. sevelamer | 3; 1440 | Serious (due to allocation concealment) | Serious limitations | Serious limitations; (I^2^: 58%) | No serious limitations | N/A | Very low | -13 (-135 to 105) | -13 (-91 to 65) |
| Diet vs. sevelamer | 1; 60 | Not serious | No serious limitations | N/A | No serious limitations | N/A | High | 11 (-23 to 47) | 172 (20 to 323) |
| Calmag vs. sevelamer | 1; 252 | Not serious | Serious limitations | N/A | No serious limitations | N/A | Moderate | 59 (1.7 to 116) | 47 (-13 to 107) |
| Placebo vs calcium | 1;87 | Not serious | Serious limitations | N/A | No serious limitations | N/A | Moderate | 67 (3.6 to 131) | 59 (-10 to 126) |
| Lanthanum vs. calcium | 4; 190 | Serious (due to allocation concealment) | Serious limitations | No serious limitations; (I^2^: 37%) | No serious limitations | N/A | Low | 44 (9.2 to 80) | 26 (-32 to 84) |
| Diet vs. calcium | 1; 60 | Not serious | No serious limitations | N/A | No serious limitations | N/A | High | -26 (-59 to 6.8) | -29 (-62 to 4) |
| Lanthanum vs. placebo | 3; 408 | Not serious | No serious limitations | No serious limitations; (I^2^: 0%) | No serious limitations | N/A | High | 40 (29 to 50) | 40 (29 to 50) |
| Iron s placebo | 2; 235 | Not serious | No serious limitations | No serious limitations; (I^2^: 0%) | No serious limitations | N/A | High | -30 (-68 to 6.3) | -39 (-77 to 0.57) |
| Calcium and Sevelamer vs. iron | 1; 441 | Not serious | No serious limitations | N/A | No serious limitations | N/A | High | -20 (-26 to -15) | -21 (-26 to -15) |
| Common within network between-study variance 0.15 (0.34 to 0.78) | | | | | | | | |  |

**Note:** For domains “Study Limitations”, “Precision”, “Consistency”, and “Directness”: No serious limitations, Serious limitations or Very serious limitations. For the domain “Publication bias”: Not likely, Likely to exist or not applicable if the comparison has less than ten trials. Reasons are provided when rating down. All direct comparisons begin with a “High” rating.^1^Rated down two levels for imprecision;^2^We employed random effect models, ^3^Bayesian methods used, ^4^The frequentist method used. CI: Confidence interval; Crl: credible intervals; MD: mean difference; N/A: not applicable. I^2^ indicates the expected degree of change in the effect estimates due to between-study variance.

**Table L.** GRADE confidence assessments of indirect estimates per pairwise treatment comparison for parathyroid hormone when direct comparisons are available

| Treatment comparisons | First order loop available | Common comparator treatment in the dominant first order loop (in the absence of the first order loop, possible comparisons in higher order loop) | GRADE of first contributing direct comparison; name of the contributors (quality of evidence) | GRADE of second contributing direct comparison | Final GRADE of Indirect Comparison |
| --- | --- | --- | --- | --- | --- |
| Sevelamer vs. calcium | Yes | Lanthanum | Calcium lanthanum  (L) | Sevelamer lanthanum  (H) | Low |
| Placebo vs. sevelamer | Yes | Iron | Placebo vs. iron  (H) | Sevelamer vs. iron  (VL) | Very Low |
| Lanthanum vs. sevelamer | Yes | Calcium | Sevelamer placebo  (H) | Lanthanum placebo  (H) | H |
| Iron vs. sevelamer | Yes | Placebo | Sevelamer vs. placebo  (H) | Iron vs. placebo  (H) | High |
| Diet vs. sevelamer | Closed loop formed by a multi-arm trial | Not available | Not available | Not available | Not available |
| Calmag vs. sevelamer | Unconnected comparison | Not available | Not available | Not available | Not available |
| Placebo vs. calcium | Yes | Lanthanum | Calcium vs. lanthanum  (L) | Placebo vs. lanthanum  (H) | Low |
| Lanthanum vs. calcium | Yes | Sevelamer | Calcium vs. Sevelamer  (L) | Lanthanum vs. Sevelamer  (H) | Low |
| Diet vs. calcium | Yes | Sevelamer | Calcium vs. Sevelamer  (L) | Diet vs. Sevelamer  (H) | Low |
| Lanthanum vs. placebo | Yes | Calcium | Calcium vs placebo (M) | Lanthanum vs. calcium  (L) | Low |
| Iron vs. placebo | Yes | Sevelamer | Iron vs. Sevelamer  (VL) | Placebo vs. Sevelamer  (H) | Very Low |
| Calsev vs. iron | Unconnected comparison | Not available | Not available | Not available | Not available |

**Note:** A single first order loop for each pairwise comparison is used to GRADE indirect estimates. All indirect comparisons begin with the lower of the two contributing direct estimates and undergo an assessment of transitivity.; The quality of evidence rating for the indirect comparisons informing each paired comparison was the lower of the ratings of quality for the direct estimates contributing to the first or higher order loop. Abbreviations: calmag: calcium and magnesium; calsev: calcium and sevelamer; calsevlant: calcium or sevelamer or lanthanum; H: high; Low: low; M: moderate; VL: very low.

**Table M.** GRADE confidence assessments of indirect estimates per pairwise treatment comparison for parathyroid hormone when direct comparisons are unavailable^c^

| Treatment comparisons | First order loop available | Common comparator treatment in the dominant first order loop (in the absence of the first order loop, possible comparisons in higher order loop) | GRADE of first contributing direct comparison; name of the contributors (quality of evidence) | GRADE of second contributing direct comparison | Final GRADE of Indirect Comparison |
| --- | --- | --- | --- | --- | --- |
| Iron vs. calcium | Yes | Sevelamer | Calcium sevelamer  (L) | Iron sevelamer  (VL) | Very Low |
| Iron vs. lanthanum | Yes | Sevelamer | Lanthanum sevelamer  (H) | Iron sevelamer  (VL) | Very Low |
| Diet vs. placebo | Yes | Sevelamer | Diet calcium  (H) | Placebo calcium  (M) | Moderate |
| Diet vs. lanthanum | Yes | Sevelamer | Diet sevelamer  (H) | Lanthanum sevelamer  (H) | High |
| Diet vs. Iron | Yes | Sevelamer | Diet sevelamer  (H) | Iron sevelamer  (VL) | Very Low |
| Calsev vs. sevelamer | Yes | Iron | Calsev iron  (H) | Iron sevelamer  (VL) | Very Low |
| Calsev vs. calcium | No | Calcium Sevelamer (L)  Iron Sevelamer (VL)  Calcium calsev (H) | Not available | Not available | Very Low |
| Calsev vs. Placebo | Yes | Iron | Calsev iron  (H) | Placebo iron  (H) | High |
| Calsev vs. Lanthanum | No | Lanthanum placebo (H)  Placebo iron (H)  Iron calsev (H) | Not available | Not available | High |
| Calsev vs. diet | No | Diet Sevelamer (H)  Sevelamer iron (VL)  Iron calsev (H) | Not possible | Not possible | Very Low |
| Calmag vs. calcium | Yes | Sevelamer | Calcium sevelamer  (L) | Calmag sevelamer  (M) | Low |
| Calmag vs. placebo | Yes | Sevelamer | Placebo sevelamer  (H) | Calmag sevelamer  (M) | Moderate |
| Calmag vs. lanthanum | Yes | Sevelamer | Lanthanum sevelamer  (H) | Calmag sevelamer  (M) | Moderate |
| Calmag vs. Iron | Yes | Sevelamer | Iron sevelamer  (VL) | Calmag sevelamer  (M) | Very Low |
| Calmag vs. diet | Yes | Sevelamer | Diet sevelamer  (H) | Calmag sevelamer  (M) | Moderate |
| Calmag vs. calsev | No | Calmag Sevelamer (M)  Sevelamer iron (VL)  Iron calsev (H) | Not available | Not available | Very Low |

**Note:** A single first order loop for each pairwise comparison is used to GRADE indirect estimates. All indirect comparisons begin with the lower of the two contributing direct estimates and undergo an assessment of transitivity.; The quality of evidence rating for the indirect comparisons informing each paired comparison was the lower of the ratings of quality for the direct estimates contributing to the first or higher order loop. Abbreviations: calmag: calcium and magnesium; calsev: calcium and sevelamer; calsevlant: calcium or sevelamer or lanthanum; H: high; Low: low; M: moderate; VL: very low.

**Table N.** SUCRA rankings of phosphate binders

|  | Phosphorus; median (95% Crl) | Calcium; median (95% Crl) | Parathyroid hormone; median (95% Crl) |
| --- | --- | --- | --- |
| Sevelamer | 0.63 (0.25 to 0.88) | 0.57 (0.14 to 0.86) | 0.57 (0.43 to 0.71) |
| Calcium | 0.50 (0.25 to 0.88) | 0.14 (0 to 0.43) | 0.43 (0.29 to 0.57) |
| Placebo | 0 (0 to 0.25) | 0.57 (0.14 to 0.86) | 0.29 (0.14 to 0.57) |
| Lanthanum | 0.38 (0.13 to 0.88) | 0.57 (0.14 to 0.86) | 0.00 (0.00 to 0.14) |
| Iron | 0.75 (0.38 to 1) | 0.43 (0 to 0.86) | 0.86 (0.71 to 0.86) |
| Diet | 0.75 (0.25 to 1) | 1.00 (0.29 to 1) | 0.71 (0.29 to 1.00) |
| Calcium and sevelamer | 0.75 (0.13 to 1.00) | 0.14 (0 to 1) | 1 (0.86 to 1) |
| Calcium and magnesium | 0.75 (0 to 1) | 0.71 (0 to 1) | 0.14 (0 to 0.57) |

**Note:** The results of surface under the cumulative ranking curve of eight phoshate binders; CrI: Credible interval; MD: Mean difference; SUCRA: surface under the cumulative ranking curve.

**Table O**. Effectiveness outcome for mean phosphate, calcium and parathyroid hormone reductions at the end of the study period using network meta-regression analysis for trial duration

|  | Phosphate; MD (95% Crl) | Calcium; MD (95% Crl) | Parathyroid hormone; MD (95% Crl) |
| --- | --- | --- | --- |
| Meta-regression coefficient | 0.009 (-0.019 to 0.038) | 0.011 (-0.005 to 0.027) | -0.186 pg/ml (-1.847 to 1.338) |
| Calcium vs. sevelamer | 0.03 mg/dl (-0.41 to 0.46) | 0.23 mg/dl (-0.01 to 0.46) | 11.13 pg/ml (-7.06 to 30.89) |
| Placebo vs. sevelamer | 1.04 mg/dl (0.21 to 1.86) | -0.11 mg/dl (-0.47 to 0.24) | 26.95 pg/ml (-2.13 to 55.27) |
| Placebo vs. calcium | 1.01 mg/dl (0.22 to 1.82) | -0.34 mg/dl (-0.67 to 0.00) | 16.04 pg/ml (-14.50 to 42.90) |
| Lanthanum vs. sevelamer | 0.21 mg/dl (-0.56 to 0.96) | -0.05 mg/dl (-0.41 to 0.28) | 63.83 pg/ml (35.49 to 91.98) |
| Lanthanum vs. calcium | 0.18 mg/dl (-0.54 to 0.88) | -0.28 mg/dl (-0.59 to 0.03) | 53.76 pg/ml (23.53 to 79.92) |
| Lanthanum vs. placebo | -0.83 mg/dl (-1.50 to -0.17) | 0.06 mg/dl (-0.30 to 0.41) | 37.96 pg/ml (18.65 to 54.67) |
| Iron vs. sevelamer | -0.26 mg/dl (-0.94 to 0.39) | 0.10 mg/dl (-0.28 to 0.48) | -10.34 pg/ml (-26.54 to 4.48) |
| Iron vs. calcium | -0.29 mg/dl (-1.07 to 0.47) | -0.12 mg/dl (-0.57 to 0.31) | -21.87 pg/ml (-46.56 to 2.61) |
| Iron vs. placebo | -1.30 mg/dl (-2.06 to -0.58) | 0.21 mg/dl (-0.18 to 0.60) | -37.27 pg/ml (-74.29 to -2.22) |
| Iron vs. lanthanum | -0.46 mg/dl (-1.28 to 0.33) | 0.16 mg/dl (-0.30 to 0.61) | -75.15 pg/ml (-103.60 to -45.48) |

**Note:** There is no significant association between treatment effect and trial duration as credible intervals include zero; MD: Mean difference; CrI: Credible interval.

**Table P.** Assessing the global consistency in networks for each labaoraty outcome using the design-by-treatment interaction model

| Outcome | Chi-square | Degrees of freedom | P value for the global inconsistency test |
| --- | --- | --- | --- |
| Phosphate | 1.76 | 3 | 0.62 |
| Calcium | 3.77 | 6 | 0.70 |
| Parathyroid hormone | 6.35 | 6 | 0.38 |
